# SNW1 Is a Critical Regulator of Spatial BMP Activity, Neural Plate Border Formation, and Neural Crest Specification in Vertebrate Embryos

**DOI:** 10.1371/journal.pbio.1000593

**Published:** 2011-02-15

**Authors:** Mary Y. Wu, Marie-Christine Ramel, Michael Howell, Caroline S. Hill

**Affiliations:** 1 Laboratory of Developmental Signalling, Cancer Research UK London Research Institute, London, United Kingdom; 2 High-Throughput Screening Facility, Cancer Research UK London Research Institute, London, United Kingdom; The Wellcome Trust Sanger Institute, United Kingdom

## Abstract

Bone morphogenetic protein (BMP) gradients provide positional information to direct cell fate specification, such as patterning of the vertebrate ectoderm into neural, neural crest, and epidermal tissues, with precise borders segregating these domains. However, little is known about how BMP activity is regulated spatially and temporally during vertebrate development to contribute to embryonic patterning, and more specifically to neural crest formation. Through a large-scale in vivo functional screen in *Xenopus* for neural crest fate, we identified an essential regulator of BMP activity, SNW1. SNW1 is a nuclear protein known to regulate gene expression. Using antisense morpholinos to deplete SNW1 protein in both *Xenopus* and zebrafish embryos, we demonstrate that dorsally expressed SNW1 is required for neural crest specification, and this is independent of mesoderm formation and gastrulation morphogenetic movements. By exploiting a combination of immunostaining for phosphorylated Smad1 in *Xenopus* embryos and a BMP-dependent reporter transgenic zebrafish line, we show that SNW1 regulates a specific domain of BMP activity in the dorsal ectoderm at the neural plate border at post-gastrula stages. We use double in situ hybridizations and immunofluorescence to show how this domain of BMP activity is spatially positioned relative to the neural crest domain and that of *SNW1* expression. Further in vivo and in vitro assays using cell culture and tissue explants allow us to conclude that SNW1 acts upstream of the BMP receptors. Finally, we show that the requirement of SNW1 for neural crest specification is through its ability to regulate BMP activity, as we demonstrate that targeted overexpression of BMP to the neural plate border is sufficient to restore neural crest formation in *Xenopus SNW1* morphants. We conclude that through its ability to regulate a specific domain of BMP activity in the vertebrate embryo, SNW1 is a critical regulator of neural plate border formation and thus neural crest specification.

## Abstract 2

In frog and fish embryos, SNW1 is a protein required for the spatio-temporal activity of BMP signaling necessary for neural plate border formation and specification of neural crest tissue.

## Author Summary

A subset of cells in the ectoderm of vertebrate embryos becomes the neural crest, which contributes to the bones and cartilage of the adult face. The neural crest arises in a location between the epidermis, which becomes the future skin, and the neural plate, which becomes the future central nervous system. Through our studies in both frog and fish embryos, we have discovered that the protein SNW-domain containing protein 1 (SNW1) is absolutely essential for defining the edge of the neural plate, where neural crest forms. SNW-domain containing proteins have been implicated in a variety of nuclear activities ranging from transcriptional regulation and elongation to RNA splicing. We show that SNW1 functions upstream of bone morphogenetic protein (BMP) receptors to regulate BMP activity, and is necessary for the activity of the BMP signaling pathway at the neural plate border where the neural crest is specified. In the absence of SNW1, BMP activity is reduced in this region and neural crest cells are lost. Given that SNW1 family proteins are highly conserved from nematodes to humans, SNW1's BMP regulatory function is likely conserved in other animals.

## Introduction

**Figure 1:**
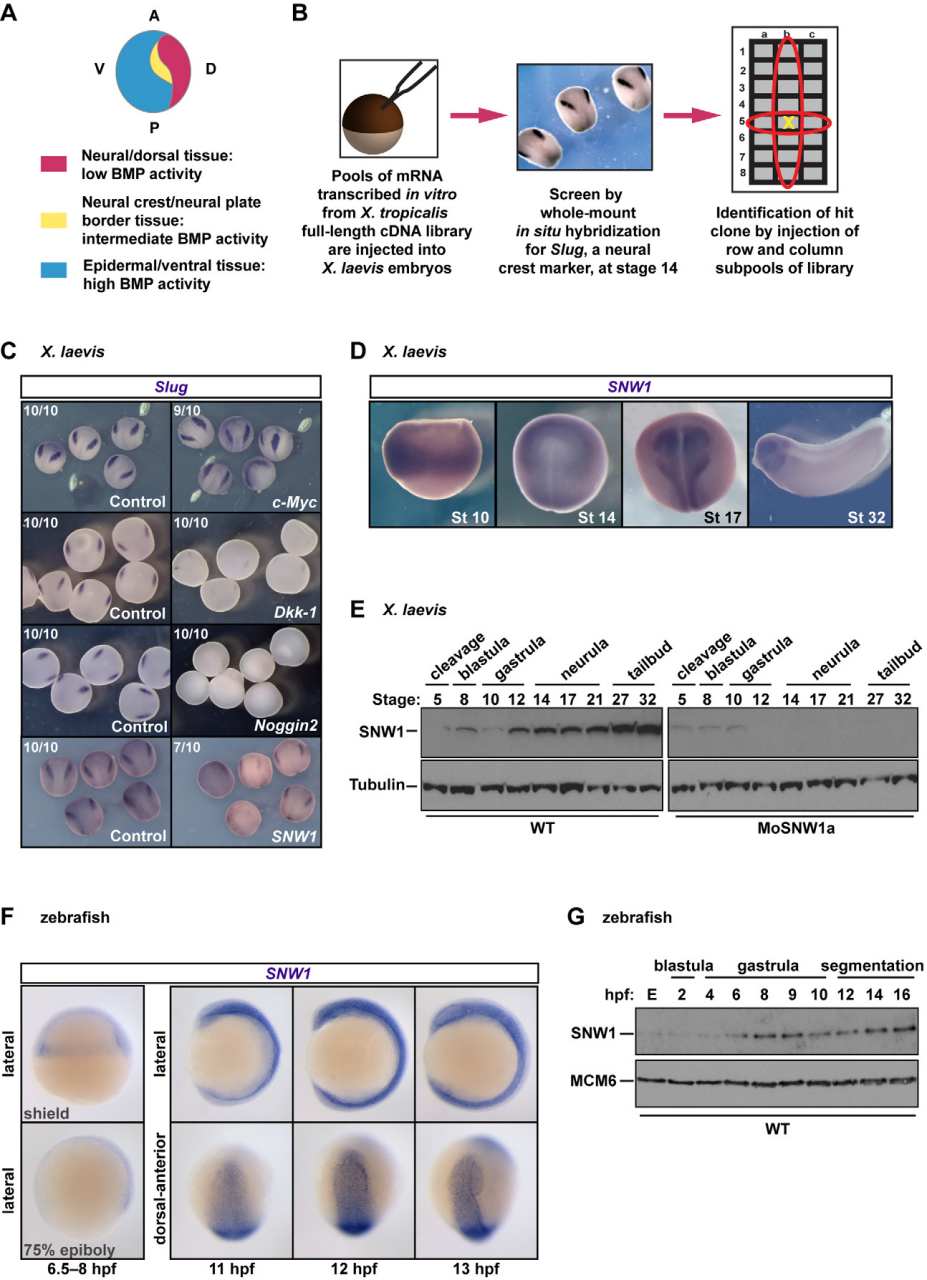
SNW1 was identified in a functional screen for neural crest fate in *X. laevis.* (A) A diagram of a stage 14 embryo depicting the classical model, which proposes that different levels of BMP signaling are required for ectodermal patterning and neural crest formation. (B) An overview of the functional screen. (C) The screen identified proteins whose overexpression modulated levels of *Slug* expression. 200 pg of mRNA expressing the *X. tropicalis* c-*Myc, Dkk-1, Noggin2,* or *SNW1* was injected at the one-cell stage, with 250 pg of *GFP* mRNA as tracer. Control embryos received the *GFP* mRNA only. Embryos were fixed at stage 14 and analyzed for *Slug* expression by WISH. The number of embryos out of the total analyzed that showed the presented staining pattern is given. (D) *X. laevis* embryos were analyzed for *SNW1* expression by WISH at the indicated stages. (E) Whole *X. laevis* embryo extracts were prepared from either wild-type (WT) embryos or embryos injected with 20 ng of a translation-blocking MO, MoSNW1a, at the one-cell stage, and analyzed by Western blotting using antibodies against SNW1 and Tubulin, the latter as a loading control. The developmental stages are indicated. (F) Zebrafish embryos were analyzed for *SNW1* expression at the indicated times. Two gastrulation stages (the shield stage and 75% epiboly) and three segmentation stages (11, 12, and 13 hpf) are shown. Lateral views are shown, and for the later stages, also a dorsal-anterior view. (G) Whole zebrafish embryo extracts were prepared from wild-type embryos at the indicated times post-fertilization and analyzed by Western blotting using antibodies against SNW1 and MCM6, the latter as a loading control. The developmental stages are indicated. E, unfertilized egg.

The development of all metazoans involves the sequential specification of restricted cell fates from pluripotent progenitors. Understanding the spatial and temporal dynamics of cell differentiation is of fundamental importance to developmental biology research, in particular, how sharp borders and domains are delineated in a field of progenitor cells. For example, the neural crest, a vertebrate-specific embryonic cell type that gives rise to many different cell types (e.g., cartilage and bones of the face, melanocytes, neurons and glia of the peripheral nervous system, and smooth muscle of the heart), is induced at a very specific domain in the developing ectoderm between the neural plate and the epidermis [1] (Figure 1A).

Patterning of the vertebrate ectoderm across its dorsal-ventral axis is known to be dependent on a gradient of bone morphogenetic protein (BMP) activity. BMP ligands are capable of functioning as morphogens, providing positional information to a field of cells depending on the strength and duration of signaling activity [2]. In *Xenopus* and zebrafish embryos, a high ventral to low dorsal gradient of BMP activity is thought to contribute to embryonic patterning. The classical model proposes that in the ectoderm, high BMP activity is required for epidermal fate, a moderate level of activity is necessary for neural plate border fates, including competency to become neural crest, and BMP inhibition is a prerequisite for neural fate [3] (Figure 1A). Analogous to the observation that gastrulating cells become competent to respond to BMP signals in a temporal fashion along the anterior-posterior axis [4], neural and neural crest fates require BMP signaling at temporally distinct periods [5],[6], in addition to the requirement for different levels of BMP activity. However, whilst these studies have demonstrated a particular requirement in time and space for BMP activity to specify certain fates, they have provided little insight into how the BMP activity may change spatially and temporally to provide the means for these developmental events to occur, or how this is regulated. Indeed, while the BMP activity gradient has been visualized in gastrulating *Xenopus* and zebrafish embryos [4],[7], no comprehensive visualization of BMP activity domains at post-gastrula stages has been reported in *Xenopus* or zebrafish. The ability to directly observe how BMP activity is remodeled at different stages will help determine how this contributes to the specification of particular cell fates and the formation of sharp tissue boundaries, as well as clarifying which BMP ligands are likely to contribute to BMP activity in specific tissues.

Here, we report the identification of a novel regulator of BMP activity in the vertebrate embryo, SNW1, in a large-scale functional screen in *Xenopus* embryos for neural crest fate. The expression of *SNW1* is enriched in dorsal tissues during neurula stages in both *Xenopus* and zebrafish embryos. Depletion of SNW1 in both organisms leads to a loss of neural crest, concomitant with an expansion of the neural plate and loss of the sharp neural plate border. We have established that dorsally expressed SNW1 is critical for neural crest formation and that the effects of SNW1 occur independently of mesoderm formation. In addition to its effect on neural crest specification, we have shown that SNW1 is necessary for BMP activity during neurula stages. More specifically, in the absence of SNW1, a specific domain of BMP activity is lost in the ectoderm, adjacent to the neural plate. Experiments in mammalian cell culture and *Xenopus* embryo explants indicate that SNW1 does not directly affect the core BMP signaling pathway, suggesting that SNW1 must function upstream of the BMP receptors. Finally the proximity/overlap of the SNW1 expression domain, neural crest cells, and the BMP activity domain prompted us to test whether SNW1 affects neural crest formation via its ability to regulate BMP activity. Indeed, targeted overexpression of BMP2b was able to rescue the neural crest phenotype of *Xenopus SNW1* morphants. Thus, SNW1 is a critical factor that is required for a specific BMP activity domain in the ectoderm in order to specify neural crest fate during neurulation.

## Results

### Identification of *SNW1* in a Functional Overexpression Screen for Neural Crest Regulators

To discover how a new cell population can be specified at a defined location in a developing vertebrate embryo we performed a large-scale in vivo functional screen in *Xenopus laevis* for neural crest fate, which occurs at the neural plate border (Figure 1A and 1B). A *Xenopus tropicalis* full-length cDNA library was used as a template for transcribing mRNA in vitro. The mRNAs were then pooled and injected into *Xenopus* embryos for overexpression. Embryos were fixed at the early neurula stage 14 and subjected to whole-mount in situ hybridization (WISH) for the neural crest marker *Slug*. Positive pools were then deconvoluted by injection of subpools and again assessed by WISH for *Slug* until single clones were isolated. We found a total of 14 proteins whose overexpression altered the levels of the neural crest marker *Slug*, and the screen was validated by the fact that some of the positive hits were known modulators of signaling pathways required for neural crest fate, or factors previously identified as neural crest regulators. Examples were c-*Myc,* whose overexpression resulted in increased *Slug* staining [8], and the Wnt antagonist *Dickkopf1* (*Dkk-1*) and the BMP antagonist *Noggin2* [1],[9],[10], both of which repressed neural crest fate (Figure 1C). One unexpected hit was *SNW1*, which substantially inhibited *Slug* staining when overexpressed (Figure 1C). SNW1, which is a nuclear protein, has been variously implicated in transcription regulation in response to several different signals [11]–[13], and in transcriptional elongation through its ability to interact with pTEFb [14], and in yeast it is thought to be involved in pre-mRNA splicing [15]. It is extremely well conserved at the amino acid sequence level throughout its entire sequence from *Caenorhabditis elegans* to humans (Figure S1).

### *SNW1* Is Ubiquitously Expressed during Gastrulation, but Is Enriched in the Dorsal Ectoderm and Mesoderm during Neurulation

Semi-quantitative reverse transcription PCR (RT-PCR) indicated that *SNW1* is expressed at all stages of *Xenopus* development examined (Figure S2A), and the presence of *SNW1* mRNA in unfertilized eggs suggests that it is maternally deposited during oogenesis. Quantitative PCR (qPCR) on dorsal and ventral halves of *Xenopus* embryos confirms that *SNW1* transcripts are present in both halves of the embryo (Figure S2B), but WISH for *SNW1* mRNA at two neurula stages (stage 14 and 17) reveals that *SNW1* expression is specifically enriched on the dorsal side, in the neural plate region (Figure 1D). Western blotting demonstrated that SNW1 protein levels were relatively low up to the end of gastrulation (stage 12), at which point the levels increased and continued to do so through the rest of development to stage 32 (Figure 1E). The 61-kDa SNW1 band disappears when SNW1 is knocked down by injection of a translation-blocking antisense morpholino (MO), MoSNW1a (Figures 1E and S3A). Interestingly, this MO efficiently reduced SNW1 protein levels from stage 12 onwards (Figure 1E), suggesting that the protein detected prior to this stage is maternally deposited and thus resistant to the MO, whereas the protein detectable after stage 12 is zygotically produced.

To investigate whether *SNW1* expression was conserved in another vertebrate species, we cloned zebrafish *SNW1* and examined its expression pattern. WISH on the naturally translucent zebrafish embryos provided higher resolution in terms of identifying the tissues where *SNW1* mRNA is enriched. During gastrula stages, *SNW1* is expressed ubiquitously. By 11 h post-fertilization (hpf), *SNW1* is detected ubiquitously in the embryo, except in the yolk-associated tissue. It appears to be enriched anteriorly on the dorsal side and is expressed in both the ectoderm and mesoderm (Figure 1F). The same expression pattern persists until 13 hpf. Consistent with what we observed in *Xenopus* embryos, the SNW1 protein is deposited maternally in zebrafish embryos and increases in levels from 6 hpf (Figure 1G).

### SNW1 Is Absolutely Required for Neural Crest Induction in *Xenopus*

**Figure 2:**
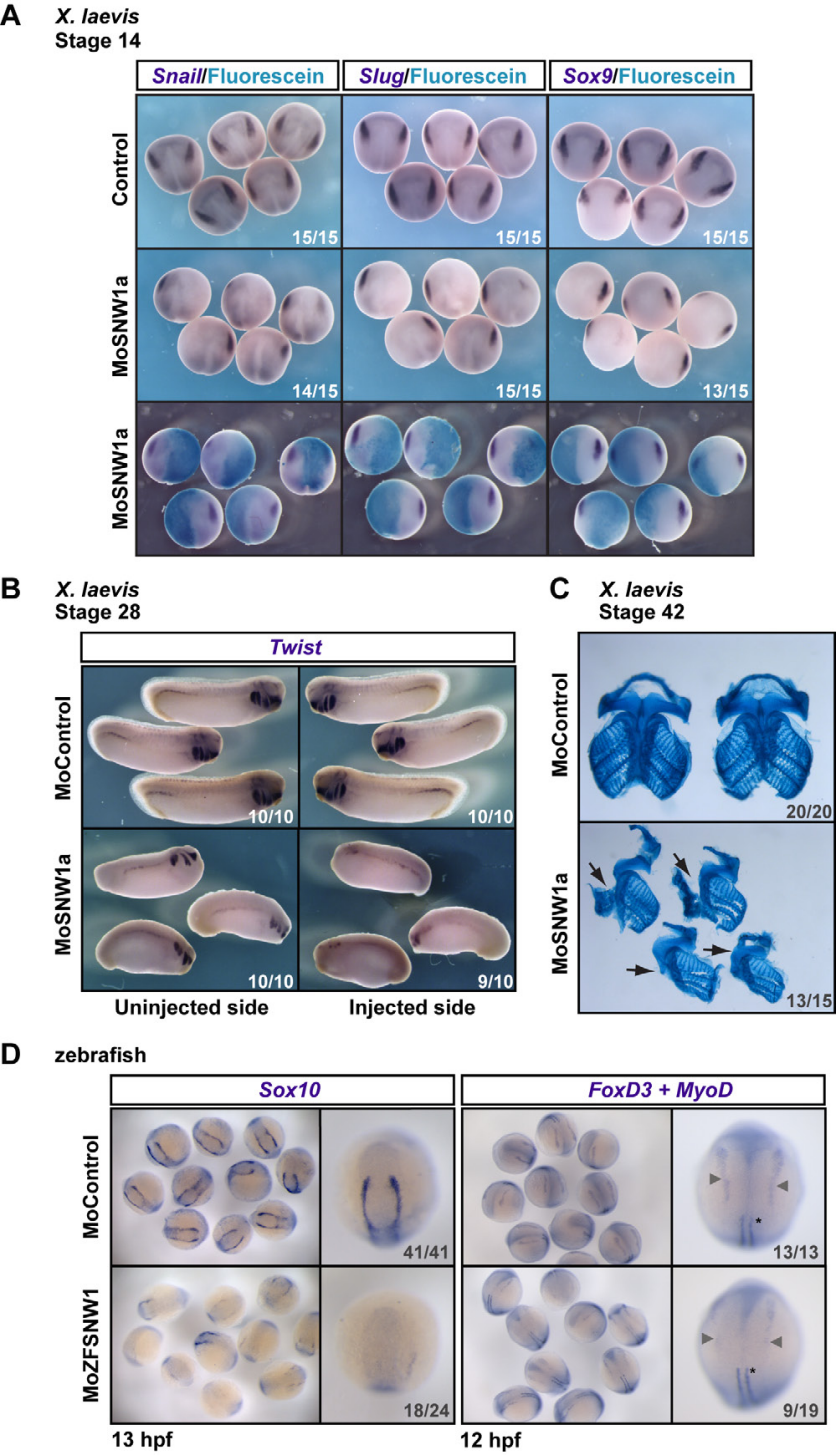
SNW1 knockdown inhibits neural crest induction. (A) Injection of MoSNW1a (Figure S3) results in the loss of neural crest markers *Snail, Slug*, and *Sox9* on the injected side at stage 14. MoSNW1a (20 ng) was co-injected with Fdx as a tracer in one cell of a two-cell *Xenopus* embryo. The tracer was detected using an anti-fluorescein antibody. Control embryos were uninjected. (B) Two-cell *Xenopus* embryos were injected in one cell with either 20 ng of control MO (MoControl) or 20 ng of MoSNW1a. WISH was performed at stage 28 using a probe against *Twist*. (C) *Xenopus* embryos were injected with control MO or MoSNW1a as in (B). At stage 42 the facial cartilage was stained with alcian blue and then dissected. The injected side is indicated with an arrow. (D) Zebrafish embryos were injected with 15 ng of either a control MO or MoZFSNW1 and fixed at 13 hpf for WISH using a probe against the neural crest marker *Sox10,* or at 12 hpf for WISH with probes against *FoxD3* and the adaxial and paraxial mesoderm marker *MyoD*. A group of embryos is shown, as well as a representative single embryo. The neural crest cells are marked by grey arrowheads, and the MyoD-postive cells by an asterisk (righthand panels). For the *MyoD* staining, only the adaxial mesoderm is visible, and this is not affected by SNW1 depletion. In all cases the number of embryos out of the total analyzed that showed the presented staining pattern or phenotype is given.

SNW1 was isolated as a neural crest repressor in the overexpression screen, so we expected that depletion of SNW1 in embryos would induce more neural crest. Surprisingly, injection of MoSNW1a into one cell of a two-cell *Xenopus* embryo resulted in a dramatic loss of the neural crest markers *Snail, Slug,* and *Sox9* [16],[17] on the injected side (Figure 2A). To check that the loss of *Snail, Slug,* and *Sox9* staining reflected an inhibition of neural crest fate and not merely a delay, we investigated the effect of SNW1 knockdown later in development, when the neural crest cells have migrated and invaded the branchial arches [1], using *Snail* and *Twist* as markers [18]. At stage 28 the amount of *Twist*-positive neural crest cells in morphant embryos was severely reduced on the injected side compared with the uninjected side and both sides of control embryos (Figure 2B). Similarly, a dramatic loss of *Snail*-stained neural crest cells was observed in MoSNW1a-injected embryos, and the neural crest cells that were induced did not migrate normally (Figure S3D). This was also evident when a different MO was used (MoSNW1b) (Figure S3A, S3C, and S3D).

We also examined neural crest derivatives at later stages of development. Cranial neural crest cells differentiate into cartilage and bones of the face, among other cell types [19]. Examination of branchial cartilage formation using alcian blue staining of MoSNW1a-injected embryos at the tadpole stages indicated that where SNW1 was depleted in half the embryo, the cartilage of the face did not form correctly (Figure 2C). Closer examination of the tadpoles demonstrated that non-neural-crest derivatives of the face formed correctly, such as the eyes (Figure S3E) and the blood vessels (data not shown).

Given that both overexpression and depletion of SNW1 had the same inhibitory effect on neural crest induction in *Xenopus*, it was important to determine whether *X. tropicalis SNW1* mRNA could rescue *X. laevis* SNW1 depletion. The five mismatches between the MoSNW1a sequence and the *X. tropicalis SNW1* sequence (Figure S3A) are sufficient to protect the *X. tropicalis* mRNA from silencing by the MO (Figure S3B). *Xenopus* embryos injected with the MO alone or with *X. tropicalis SNW1* mRNA alone showed reduced expression of *Slug* and *Sox9*. However, injection of the MO with the *X. tropicalis SNW1* mRNA restored both *Slug* and *Sox9* expression (Figure S4A), confirming the specificity of both the overexpression and knockdown phenotypes. Interestingly, opposite effects of knockdown and overexpression on tailbud stage morphology were observed (Figure S4A, bottom row). Depletion of SNW1 resulted in dorsalized embryos that curled downwards because of more tissue on the dorsal side, whereas overexpression of SNW1 resulted in slightly ventralized embryos with smaller heads and anterior structures. This suggests that distinct mechanisms account for the repression of neural crest when SNW1 is overexpressed versus when it is depleted, which is discussed in more detail below.

### The Role of SNW1 in Neural Crest Fate Is Evolutionarily Conserved

To investigate whether the requirement of SNW1 for neural crest fate was conserved in zebrafish we designed an antisense MO, MoZFSNW1, to block splicing (Figure S3A). Despite being injected within the first hour post-fertilization, this MO is effective only after 8 hpf, suggesting it inhibits only zygotic transcripts, analogous to the situation with the *Xenopus SNW1* MOs. Knockdown of SNW1 in zebrafish using MoZFSNW1 inhibited neural crest fate as demonstrated by *Sox10* and *FoxD3* staining (Figure 2D). A dorsalized-like phenotype was also observed when SNW1 was depleted in zebrafish embryos with MoZFSNW1 (Figure S4B). This phenotype, which was characterized by loss of ventral tail fin tissues, and loss of posterior structures including the most posterior somites, was partially rescued by overexpression of *X. laevis SNW1* (Figure S4C). Thus, the requirement for SNW1 for neural crest fate is conserved in zebrafish.

### SNW1 Is Required in the Dorsal Ectoderm for Neural Crest Fate

**Figure 3:**
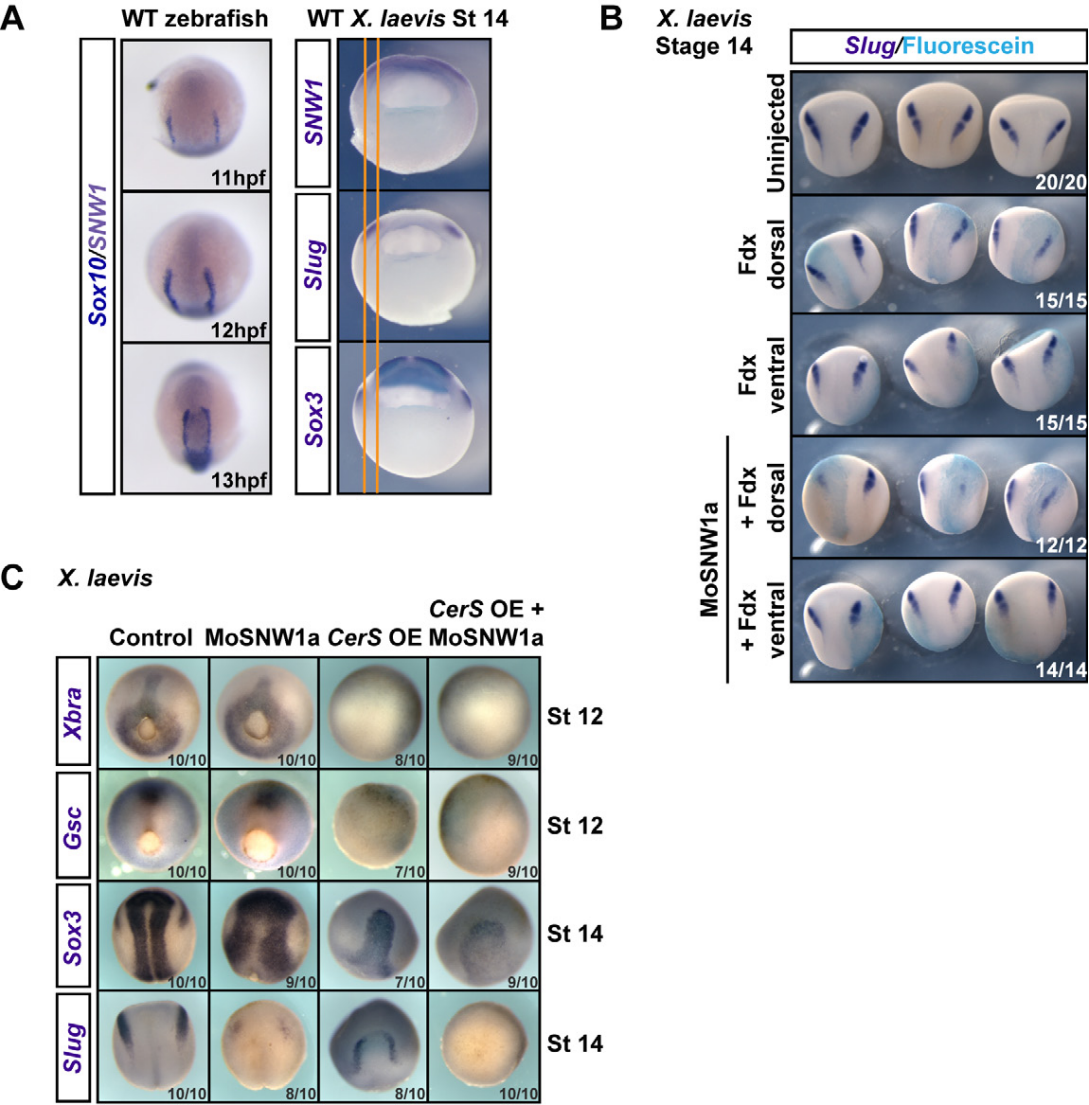
Dorsally expressed SNW1 is required for neural crest specification, and this is independent of mesoderm. (A) Left panels. Wild-type (WT) zebrafish embryos at the indicated times post-fertilization were doubly stained for the neural crest marker *Sox10* (dark blue) and *SNW1* (grey-purple), which is localized in the neural plate (see Figure 1F). Right panels. Wild-type stage 14 *Xenopus* embryos were stained for either *SNW1*, the neural crest marker *Slug* or the neural plate marker *Sox3*. The orange lines mark the edges of the neural crest staining to enable direct comparison of expression domains. (B) One dorsal or one ventral blastomere of four-cell *Xenopus* embryos was injected with Fdx with or without 10 ng of MoSNW1a, as indicated. At stage 14 the embryos were stained for *Slug* and fluorescein. (C) *Xenopus* embryos were either uninjected (control), injected with 20 ng of MoSNW1a at the one-cell stage, injected with 250 pg of *CerS* mRNA into all four blastomeres at the four-cell stage, or injected with MoSNW1a at the one-cell stage followed by *CerS* mRNA at the four-cell stage. WISH was carried out for the mesoderm markers *Xbra* and *Gsc* at stage 12, or the neural plate marker *Sox3* and the neural crest marker *Slug* at stage 14. OE, overexpression. In all cases the number of embryos out of the total analyzed that showed the presented staining pattern is given.

As demonstrated by qPCR on dorsal and ventral halves of *Xenopus* embryos, *SNW1* is expressed in both of these domains (Figure S2B). However, in situ staining in *Xenopus* and zebrafish embryos revealed that *SNW1* mRNA is enriched in the dorsal ectoderm and mesoderm (Figure 1D and 1F). Specifically, in both organisms the enriched domain of expression in the ectoderm overlaps the neural plate as well as the neural crest (Figure 3A). To determine whether it was the dorsally expressed *SNW1* that was required for neural crest specification we targeted the injection of MoSNW1a to either a dorsal or ventral quadrant of *Xenopus* embryos at the four-cell stage. Only depletion of SNW1 on the dorsal side, where its expression is enriched, affected neural crest fate (Figure 3B).

Since *SNW1* is also enriched in dorsal mesoderm, it was important to establish that the effects of SNW1 depletion on neural crest fate were direct and not the result of perturbing earlier mesoderm induction or gastrulation movements, both of which could influence neural crest specification [1]. Depletion of SNW1 had no effect on the mesoderm marker *Xbra*, nor on the dorsal and ventral mesoderm markers *Goosecoid (Gsc)* and *Sizzled (Szl),* respectively, as examined by WISH at stage 12 (Figure S5A). Knockdown of SNW1 also had no effect on mesoderm induction or convergence-extension movements in an animal cap assay [20] (Figure S5B). Moreover, in zebrafish, depletion of SNW1 had no effect on the mesoderm markers *No tail a*, *Even-skipped-like 1, Cdx4* (Figure S5C), or *MyoD* (Figure 2D).

To demonstrate that the loss of neural crest fate upon depletion of SNW1 is a direct effect on ectodermal patterning, we inhibited mesoderm induction by overexpressing Cerberus short (CerS) in *Xenopus* embryos. Full-length Cerberus inhibits Nodal, Wnt, and BMP signaling, whereas the N-terminally truncated form, named CerS, inhibits only Nodal signaling and hence mesoderm formation [21]. The expression of the mesoderm markers *Xbra* and *Gsc* was completely repressed by CerS overexpression, and the embryos develop without gastrulation movements, as evidenced by the lack of a blastopore (Figure 3C). Despite a lack of mesoderm formation, the neural plate and neural crest cells are induced, as seen by robust staining for *Sox3* and *Slug,* respectively, in embryos overexpressing CerS (Figure 3C, lower two rows). Importantly, injection of MoSNW1a expands the expression domain of *Sox3*, consistent with the dorsalized morphology observed previously (Figure S4A), and represses the expression of *Slug* in both wild-type and CerS-overexpressing embryos (Figure 3C). These results indicate first that, contrary to what was previously thought [19], neural crest fate appears to be induced in *Xenopus* embryos in the absence of mesoderm, and second that the effect of SNW1 depletion on neural crest fate occurs entirely by perturbing ectoderm patterning.

### SNW1 Is Required for Formation and Sharpening of the Neural Plate Border

**Figure 4:**
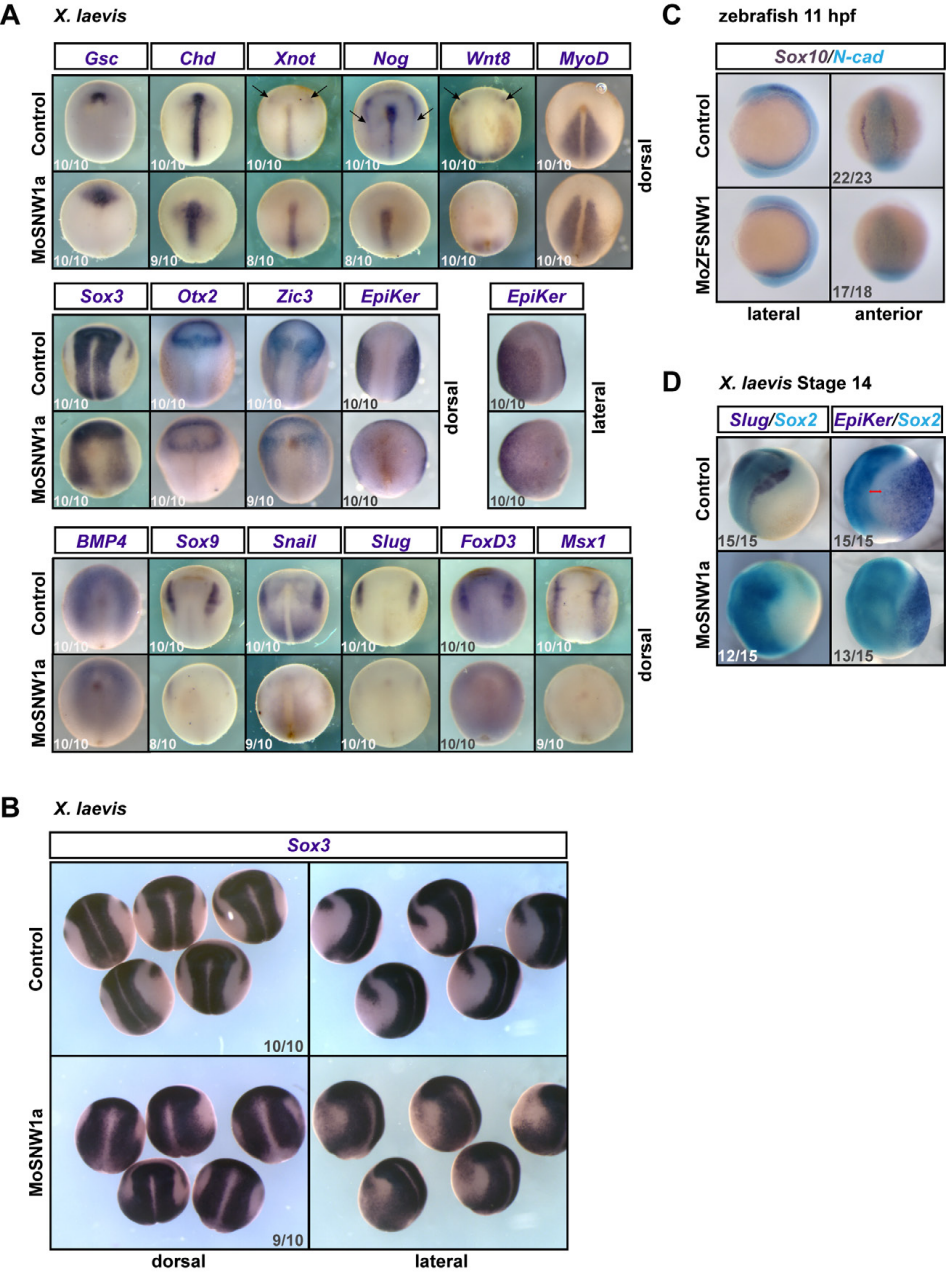
SNW1 is essential for specification of the border between neural and non-neural ectoderm. (A) *Xenopus* embryos were either uninjected (control) or injected with 20 ng of MoSNW1a at the one-cell stage. WISH was carried out for the dorsal mesoderm marker *Goosecoid* (*Gsc*), the dorsal midline markers *Chordin* (*Chd*), *Xnot*, and *Noggin* (*Nog*), the paraxial mesoderm markers *Wnt8* and *MyoD*, the neural plate marker *Sox3*, the anterior neural marker *Otx2*, the anterior neural and neural plate border marker *Zic3*, the epidermal marker *Epidermal keratin* (*EpiKer*), and the neural plate border markers *BMP4*, *Sox9, Snail, Slug, FoxD3,* and *Msx1*. Arrows mark the expression of *Xnot*, *Noggin,* and *Wnt8* at the anterior neural border. For *EpiKer*, a lateral view is shown in addition to the dorsal view. In all panels anterior is to the top. (B) Lateral and dorsal views of the *Sox3* WISH are shown. Note that the sharp border of the neural plate is not formed when SNW1 is depleted. (C) Zebrafish embryos were injected with 15 ng of either a control MO (MoControl) or MoZFSNW1 and fixed at 11 hpf for double in situ hybridizations using probes against *Sox10* (purple) and the neural plate marker *N-cadherin* (*N-cad*; turquoise). Lateral and anterior views of the same embryo are shown. (D) *Xenopus* embryos were either uninjected (control) or injected with 20 ng of MoSNW1a at the one-cell stage. Double in situ hybridizations were carried out at stage 14 for *Slug* (dark blue) and *Sox2* (turquoise), or *EpiKer* (dark blue) and *Sox2* (turquoise). The red line indicates the border between the neural plate and the epidermis. In all cases the number of embryos out of the total analyzed that showed the presented staining pattern is given.

Our observation that SNW1 depletion leads to an expansion in the expression domain of *Sox3,* as well as loss of neural crest, prompted us to assess how overall dorsal/ventral patterning was affected when SNW1 was knocked down. At the neurula stages, dorsal midline/notochord markers such as *Goosecoid*, *Chordin, Xnot,* and *Noggin* were strongly expressed in morphant embryos, although the notochord was visibly shortened and wider (Figure 4A), consistent with a shortening of the anterior-posterior axis seen at later stages (Figure S4A). This could be an effect on convergence-extension movements, but we do not believe that this contributes to the effect on the neural crest, since in the absence of morphogenetic movements when CerS is overexpressed, we still observed the specific effect of depleting SNW1 on the neural crest marker *Slug* (Figure 3C). Expression of *Xnot* and *Noggin* at the anterior neural plate border was also absent in the morphant embryos (Figure 4A, arrows). The paraxial mesodermal expression of *Wnt8* was lost, although the ventral mesodermal expression near the blastopore was maintained (Figure 4A, arrows), and the somitic mesodermal marker *MyoD* was not affected (Figure 4A), similar to what was seen in the fish (Figure 2D).

Next, we examined a panel of ectodermal markers, starting with the neural plate marker *Sox3*. As previously shown (Figure 3C), the domain of *Sox3* expression was expanded in *SNW1* morphants, and the border of the neural plate was very diffuse (Figure 4A and 4B). Consistent with this, the domain of *Epidermal keratin* (*EpiKer*) staining was reduced in SNW1-depleted embryos, and the sharp border between neural and non-neural tissue was lost. Examination of the anterior neural marker *Otx2* revealed little difference, except a slight expansion laterally, consistent with the observation that the neural plate is wider and that the embryos exhibit a dorsalized morphology later in development. The same was seen for *Otx2* staining in fish embryos (Figure S5C). Interestingly, in *Xenopus* the neural expression of *Zic3* parallels what was observed for *Otx2*, whereas the expression normally found at the neural plate border was fainter and more diffuse. The same was true for *BMP4*, which is normally expressed at the neural plate border but is more diffuse in *SNW1* morphants (Figure 4A).

Most importantly, neural plate border markers (*Msx1*), including neural crest markers (*Sox9, Snail, Slug,* and *FoxD3*), were completely absent in morphant embryos (Figure 4A). Thus, neural plate border specification is completely lost when SNW1 is depleted. Careful examination of *Sox3*-stained *Xenopus* morphant embryos, especially from a lateral view, reveals that the neural plate border is consistently diffuse and expanded towards the ventral side (Figure 4B). There is a clear loss of the sharp border of the neural plate. We see a similar expansion of the neural plate in zebrafish morphants and loss of neural crest as seen by *N-cadherin* and *Sox10* double in situ hybridizations (Figure 4C). Furthermore, in *Xenopus* embryos the neural plate and epidermal ectoderm appear to merge together without a distinct border between the two tissue types (Figure 4D). Without neural plate border specification and the expression of *Msx1*, neural crest cells cannot be induced [22],[23].

### SNW1 Is Required for Post-Gastrulation BMP Activity in Whole Embryos

Among factors known to be important for neural plate border specification are the transforming growth factor β (TGF-β) superfamily ligands of the BMP family [1]. The loss of neural plate border markers in the *SNW1* morphant embryos and the clear disruption of the sharp neural/non-neural border seen in embryos stained for *Sox2*/*Sox3* and *Epidermal keratin* (Figure 4B and 4D) raised the exciting possibility that SNW1 might directly affect the BMP activity in vivo that is required for dorsal/ventral patterning of the ectoderm and neural crest specification.

**Figure 5:**
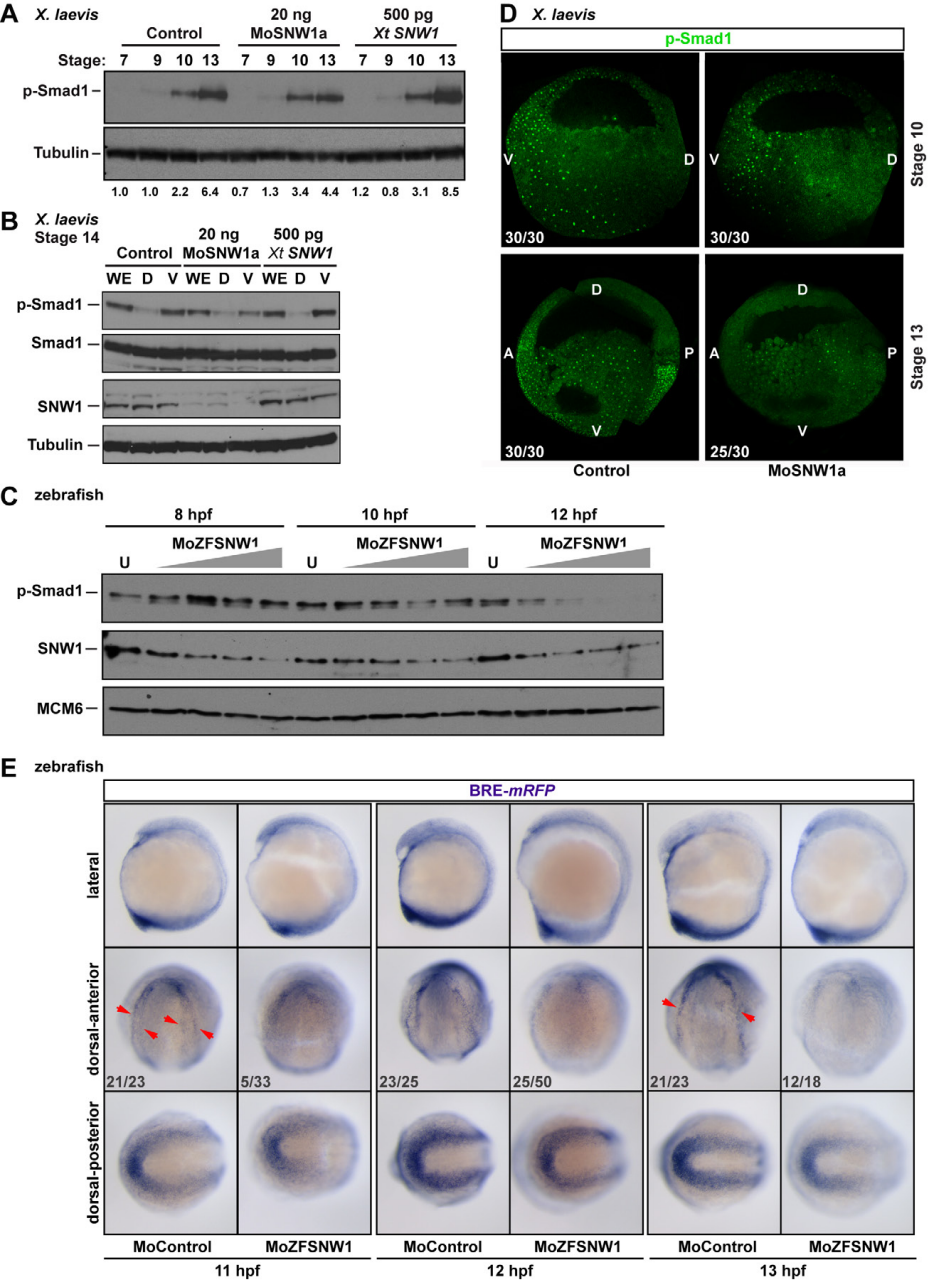
SNW1 regulates BMP activity in *Xenopus* and zebrafish embryos and is essential for BMP activity at the border between neural and non-neural tissue. (A) One-cell *Xenopus* embryos were either uninjected (control) or injected with 20 ng of MoSNW1a or 500 pg of *X. tropicalis* (*Xt*) *SNW1*. Embryos were harvested at the stages indicated, and whole embryo extracts were analyzed by Western blotting using antibodies against p-Smad1 and Tubulin, the latter as a loading control. Quantification of p-Smad1 levels relative to Tubulin is shown below the blots. (B) *Xenopus* embryos were either uninjected (control) or injected with 20 ng of MoSNW1a or 500 pg of *X. tropicalis SNW1* mRNA at the one-cell stage, and bisected at stage 14 into dorsal (D) and ventral (V) halves. Whole cell extracts were analyzed by Western blotting using antibodies against SNW1, p-Smad1, Smad1, and Tubulin, the last as a loading control. WE, whole embryo. (C) Zebrafish embryos were either uninjected (U) or injected with increasing amounts (5, 10, 15, or 20 ng) of the splice-blocking MO MoZFSNW1. Embryos were harvested at 8, 10, or 12 hpf. Whole embryo extracts were analyzed by Western blotting using antibodies against SNW1, p-Smad1, and MCM6, the last as a loading control. (D) Knockdown of SNW1 strongly reduces ventral p-Smad1 levels at stage 13, but has no effect on the p-Smad1 ventral/dorsal gradient at stage 10. Embryos that were either uninjected (control) or injected with 20 ng of MoSNW1a at the one-cell stage were fixed at either stage 10 or stage 13, sagittally bisected along the midline, and immunostained with an antibody against p-Smad1. For these panels and subsequent p-Smad1 immunostaining, the specific p-Smad1 staining is nuclear and punctate. A, anterior; D, dorsal; P, posterior; V, ventral. (E) Transgenic BRE-*mRFP* embryos were injected with 15 ng of control MO or MoZFSNW1. They were fixed at 11, 12, and 13 hpf, when WISH was performed for *mRFP*, which indicates domains of BMP activity. In each case three different views of the same embryo are shown. In control embryos, *mRFP* is expressed in the tailbud (see dorsal-posterior view) and in a horseshoe-shaped domain at the dorsal anterior at 11 hpf (red arrowheads), which becomes increasingly sharpened at 12 and 13 hpf. In *SNW1* morphants, the *mRFP* in this dorsal-anterior domain is reduced or absent. However, *mRFP* expression, and hence BMP activity, in the posterior is only slightly reduced or unchanged. In (D) and (E) the number of embryos out of the total analyzed that showed the presented staining pattern is given. In (E), the MO starts to knockdown SNW1 only from 8 hpf, and thus its effects become apparent only after 10 hpf. This explains why only a small percentage of embryos at 11 hpf shows the presented phenotype. By 13 hpf, when the MO effectively knocks down SNW1, 66% of the embryos show the phenotype.

BMP signaling leads to phosphorylation of the intracellular signal transducer Smad1 [2] and thus phosphorylated Smad1 (p-Smad1) can be used as a readout of BMP activity. BMP activity is first detected in developing *Xenopus* embryos at stage 9, and its activity increases through gastrulation and early neurulation as indicated by the level of p-Smad1 detected in whole embryo lysates (Figure 5A). At stage 13 (early neurula), SNW1-depleted embryos exhibited lower p-Smad1 compared to control embryos, and SNW1 overexpression resulted in higher levels of p-Smad1 compared to control embryos (Figure 5A). This would account for the opposite phenotypes seen when SNW1 is knocked down versus overexpressed (Figure S4A).

The significant stage-specific decrease in p-Smad1 levels in *SNW1*-morphant *Xenopus* embryos prompted us to examine how BMP activity is affected spatially. When stage 14 *Xenopus* embryos were bisected into dorsal and ventral halves, the majority of p-Smad1 detected in whole embryo lysates was derived from the ventral halves of embryos (Figure 5B). This ventral BMP signaling was substantially decreased in the morphant embryos, but slightly increased when SNW1 was overexpressed (Figure 5B). Similarly, knockdown of SNW1 in zebrafish embryos led to a dose-dependent decrease in p-Smad1 levels from 12 hpf (4–5 somites) (Figure 5C).

To visualize the spatial differences in BMP activity detected by Western blotting (Figure 5B) and determine how they are affected by SNW1 depletion, we initially used immunostaining for p-Smad1 in fixed *Xenopus* embryo halves to directly observe BMP activity in vivo. Consistent with the fact that MoSNW1a does not deplete SNW1 protein prior to stage 12 (Figure 1E), there was no effect on p-Smad1 staining in stage 10 embryos that were bisected sagittally. Thus, the initial ventral to dorsal gradient formed normally in morphant embryos. However, at stage 13, when the BMP activity gradient has changed spatially, a clear loss of the nuclear p-Smad1 staining was evident on the ventral side of morphant embryos and in the anterior (Figure 5D), consistent with the Western blotting data.

We have already demonstrated that *SNW1* expression is enriched on the dorsal side of the embryo and that the enhanced expression on this side of the embryo is responsible for its effects on neural crest fate (Figures 1D and 3B). Therefore, the reduction of p-Smad1 on the ventral side cannot explain the effects on neural crest fate. This suggested that there must be a more localized domain of BMP activity that specifically influences neural crest specification. To gain better resolution when examining BMP activity in vivo, we generated a zebrafish reporter transgenic line for BMP signaling. This line contains a stably integrated *monomeric red fluorescent protein* (*mRFP*) reporter driven by BMP responsive elements (BRE). An initial characterization of this BRE-*mRFP* line is shown in Figure S6. WISH for *mRFP* in control embryos at 11–13 hpf reveals known domains of BMP activity, such as the tailbud (Figure 5E; see dorsal-posterior views), where high p-Smad1 staining has been reported [24],[25]. In the anterior we detect a novel horseshoe-shaped domain of BMP activity at the border region between neural and non-neural ectoderm. This domain progressively sharpens from 11 hpf to 13 hpf (see red arrows, Figure 5E). In *SNW1* morphant embryos, *mRFP* levels in this domain are significantly reduced. Very interestingly, *mRFP* is most strongly reduced at the lateral edges of the horseshoe-shaped domain in *SNW1* morphants, whereas the tailbud and the most anterior neural plate border activities are not significantly affected (Figures 5E and S7B).

We examined which BMP ligands may be responsible for this localized activity at the neural plate border by carrying out in situ staining for *BMP2b*, *BMP4*, and *BMP7* in zebrafish embryos. *BMP2b* in zebrafish, which is functionally equivalent to *BMP4* in *Xenopus* [26], is specifically expressed at this region, whereas zebrafish *BMP4* is mainly expressed in the anterior prechordal plate and tailbud, and zebrafish *BMP7* is predominantly expressed in the endoderm (Figure S7). Importantly, *BMP2b* transcripts were unaltered in *SNW1* morphants, indicating that SNW1 must act downstream of *BMP2b* transcription (Figure S7B).

The BRE-*mRFP* zebrafish line provided us with increased resolution when observing BMP activity in vivo. This prompted us to use immunostaining of p-Smad1 on transversely bisected *Xenopus* embryos to see whether, indeed, BMP activity at the neural plate border is conserved and whether this localized BMP activity requires SNW1. Fluorescein dextran (Fdx) with or without MoSNW1a was injected into one cell at the two-cell stage (Figure S8). In embryos injected with Fdx alone, p-Smad1 could be detected at the region of the embryo that corresponds to the neural plate border on both the injected and uninjected sides. In embryos injected with Fdx and MoSNW1a, p-Smad1 could be detected only on the uninjected side of the embryo (see enlarged images, Figure S8). Thus, the requirement for SNW1 for BMP activity at the end of gastrulation is conserved in the two species examined. Furthermore, SNW1 is specifically required for the localized BMP activity found at the lateral edges of the neural plate.

### Neural Crest Cells Form in a Distinct Region at the Intersection of the Dorsally Enriched *SNW1* Expression Domain and the BMP Activity Domain

**Figure 6:**
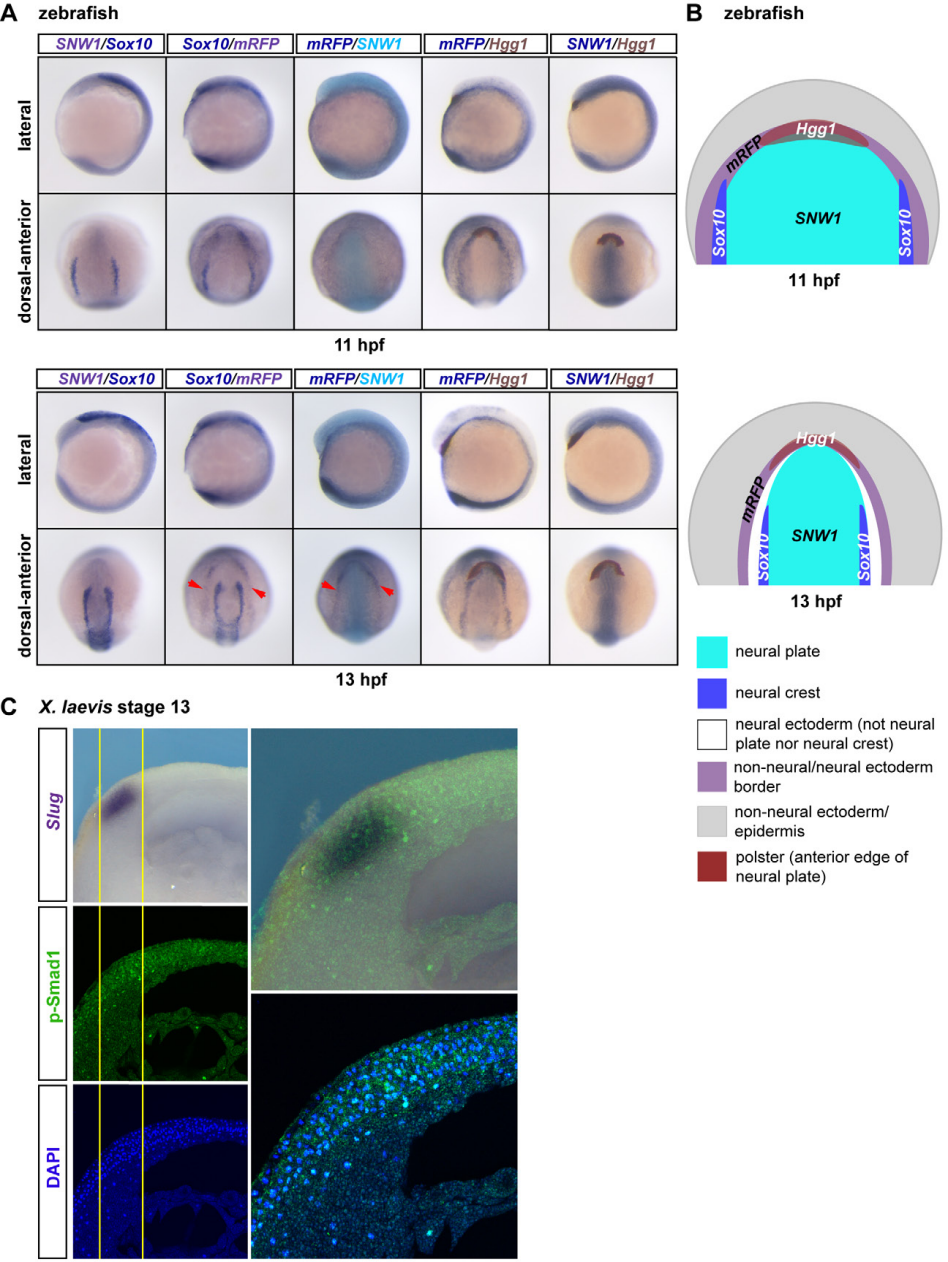
In post-gastrula zebrafish and *Xenopus* embryos the neural crest domain overlaps with the dorsal-anterior domain of BMP activity. (A) At early segmentation stages of zebrafish development the domains of expression of *SNW1* and *Sox10* partially overlap with the anterior “horseshoe” domain of BMP activity. Transgenic BRE-*mRFP* zebrafish embryos were stained for *SNW1* (grey-purple) and *Sox10* (dark blue), *Sox10* (dark blue) and *mRFP* (grey-purple), *mRFP* (dark blue) and *SNW1* (turquoise), *mRFP* (dark blue) and *Hgg1* (maroon), or *SNW1* (dark blue) and *Hgg1* (maroon) at 11 hpf and 13 hpf. *mRFP* staining indicates regions of BMP activity. (B) A diagram representing the staining patterns of the four markers is shown. At 11 hpf, domains of expression of *SNW1, Sox10,* and *mRFP* partially overlap at the lateral edges of the dorsal-anterior horseshoe domain. However at 13 hpf, it is clear that although the neural crest marker *Sox10* is still adjacent to the domain of *SNW1* expression in the neural plate, the horseshoe *mRFP* expression domain (red arrowheads in [A]) is separated from the *Sox10* and *SNW1* expression domains. (C) Wild-type stage 13 *Xenopus* embryos were bisected transversely through the neural crest region. The anterior half was immunostained with an antibody against p-Smad1, and the posterior half was used for WISH against *Slug*. The embryos were then imaged along the plane of bisection and overlayed. The yellow lines outline the boundaries of detected p-Smad1 staining at the neural plate border, which overlaps with the *Slug*-positive neural crest cells.

We have demonstrated that SNW1 is required for neural crest fate, and have characterized the essential role of SNW1 in regulating BMP activity post-gastrulation. To find the link between these two activities of SNW1, we further exploited the BRE-*mRFP* fish line to visualize how *SNW1* expression, neural crest cells, and the neural plate border BMP activity relate to each other spatially over time. Double in situ hybridization was carried out on BRE-*mRFP* embryos at 11 and 13 hpf. At 11 hpf, *Sox10*-positive neural crest cells and the enriched *SNW1* expression domain in the neural plate overlap (Figure 6A and 6B). Neural crest cells also overlap with the broad horseshoe domain of BMP activity, as detected by in situ staining for *mRFP* (Figure 6A and 6B). Double in situ staining for *SNW1* and *mRFP* demonstrates that at 11 hpf, there is no detectable separation between these domains (Figure 6A and 6B). Thus, neural crest cells appear to form where *SNW1* expression and BMP activity meet. By 13 hpf, when the broad domain of BMP activity has sharpened, the embryos have undergone elongation, and the neural tube has started forming, there is clear separation between the *SNW1* expression domain, including neural crest cells, and the domain of BMP activity (Figure 6A and 6B).

We carried out a similar analysis in *Xenopus* embryos by transversely bisecting embryos and carrying out WISH for *Slug* on one half, and immunostaining for p-Smad1 on the other half. Overlay of the p-Smad1 staining on the *Slug* staining reveals that the domain of BMP activity at the neural plate border overlaps with the neural crest cells (Figure 6C).

### SNW1 Is Not a Core Component of the Intracellular BMP Signal Transduction Pathway and Must Function at the Level of, or Upstream of, the BMP Receptors

**Figure 7:**
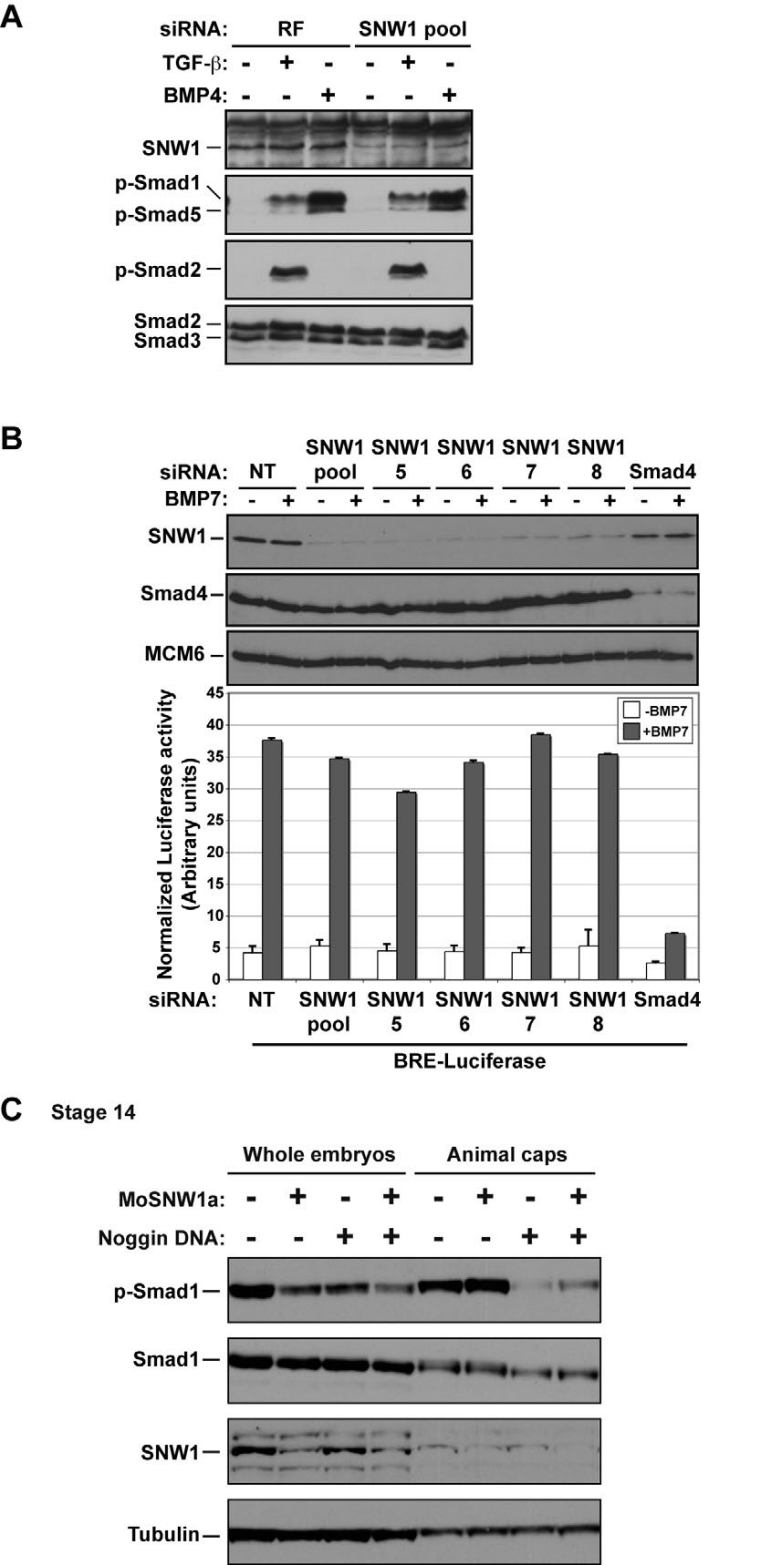
SNW1 does not function downstream of the receptors in the BMP pathway in tissue culture cells or in *Xenopus* animal cap explants. (A) Knockdown of SNW1 had no effect on the levels of p-Smad1/5 or p-Smad2 in response to ligand stimulation. SNW1 was depleted in MDA-MB-231 cells using an siRNA pool containing four individual duplexes that specifically target the human *SNW1* mRNA. 72 h after siRNA transfection, cells were either left uninduced or induced for 1 h with 20 ng/ml BMP4 (R&D Systems) or 2 ng/ml TGF-β (PeproTech). Whole cell extracts were analyzed by Western blotting using antibodies against SNW1, p-Smad1/5, p-Smad2, and Smad2/3, the last serving as a loading control. In this gel system p-Smad1 and p-Smad5 are resolved into two bands. RiscFree (RF) siRNA is a non-targeting duplex used as a control. (B) siRNA-mediated knockdown of SNW1 in mammalian cells does not affect a BMP-responsive reporter activity. An MDA-MB-231 cell line stably expressing TK-Renilla and BRE-Luciferase was transfected with a non-targeting siRNA (NT), an siRNA pool against *SNW1*, the four individual siRNAs that make up the pool, or an siRNA against *Smad4*. 72 h after transfection, cells were induced with 200 ng/ml BMP7 (PeproTech). Samples were taken for Western blotting after 1 h and for Luciferase/Renilla assays after 8 h. The efficiency of knockdown is demonstrated in the Western blot shown. (C) SNW1 depletion affects BMP activity only in whole embryos and not in animal caps. Embryos were either uninjected or injected at the one-cell stage with 20 ng of MoSNW1a or 10 pg of a plasmid expressing Noggin. Animal caps were cut at stage 8.5 and cultured until sibling embryos had reached stage 14. Whole cell extracts from whole embryos or the animal caps were analyzed by Western blotting using antibodies against p-Smad1, Smad1, SNW1, and Tubulin, the last serving as a loading control.

Since two studies have previously suggested a role for SNW1 in BMP- and TGF-β-dependent transcription [11],[27], and mammalian SNW1 was originally discovered in a two-hybrid screen for proteins that interact with the transcriptional repressor of TGF-β superfamily signaling pathways, Ski [28],[29], we investigated whether SNW1 might elicit its effects on neural crest fate by functioning intracellularly in TGF-β superfamily signal transduction pathways. We decided to use MDA-MB-231 cells for these assays as they express the necessary receptors and were previously shown to have a robust response to both TGF-β and BMPs at the levels of Smad phosphorylation and transcriptional output [30]. Small interfering RNA (siRNA) depletion of SNW1 in MDA-MB-231 cells, however, had no effect on the levels of phosphorylated Smad1/5 or Smad2 in response to TGF-β or phosphorylated Smad1/5 in response to BMP4, indicating that SNW1 does not influence the BMP or TGF-β signaling pathways at the level of Smad activation in these cells (Figure 7A). Furthermore, SNW1 depletion using either the siRNA pool or individual siRNAs had no effect on the induction of a BMP-dependent reporter, BRE-Luciferase (Figure 7B), or a TGF-β-dependent reporter, CAGA_12_-Luciferase (Figure S9A), although depletion of Smad4 reduced the activity of both of these reporters as expected [31] (Figures 7B and S9A). Moreover, despite reports of SNW1 interacting with Smad2, Smad3, and Ski when the proteins were overexpressed [11],[32], we found no evidence for such interactions for the endogenous proteins (Figure S9B), although we could confirm the previously demonstrated interaction between Ski and Smad proteins [28] in the same experiment (Figure S9B). These results argue against SNW1 functioning in either the core BMP or TGF-β pathways.

The experiments described above were performed in tissue culture cells using purified ligand. To determine whether the results hold true in *Xenopus*, we compared the effect of SNW1 knockdown on p-Smad1 levels in whole embryos at stage 14 with that in animal caps isolated at stage 8.5 then cultured to stage 14. In both of these cases the inducing ligands are endogenously produced. In whole embryos, SNW1 depletion resulted in a reduction of p-Smad1 levels equivalent to that caused by overexpression of the BMP antagonist Noggin [33] (Figure 7C). In contrast, in animal caps, depletion of SNW1 had no effect on p-Smad1 levels, whereas Noggin overexpression substantially reduced p-Smad1 (Figure 7C). SNW1 therefore affects BMP signaling only in the context of the whole embryo, and not in an isolated tissue or in cell culture, further indicating that SNW1 is not a core component of the intracellular BMP pathway.

Taking these results together with the results in the previous two sections, we conclude that SNW1 is required for BMP activity in vivo, that it plays an important role in demarcating the boundary of BMP activity at the neural plate border, and that it must act downstream of ligand transcription, but at the level of, or upstream of, the BMP receptors.

### Disruption of BMP Activity by Localized Expression of Noggin Mimics the Effect of SNW1 Depletion on Neural Crest Fate

**Figure 8:**
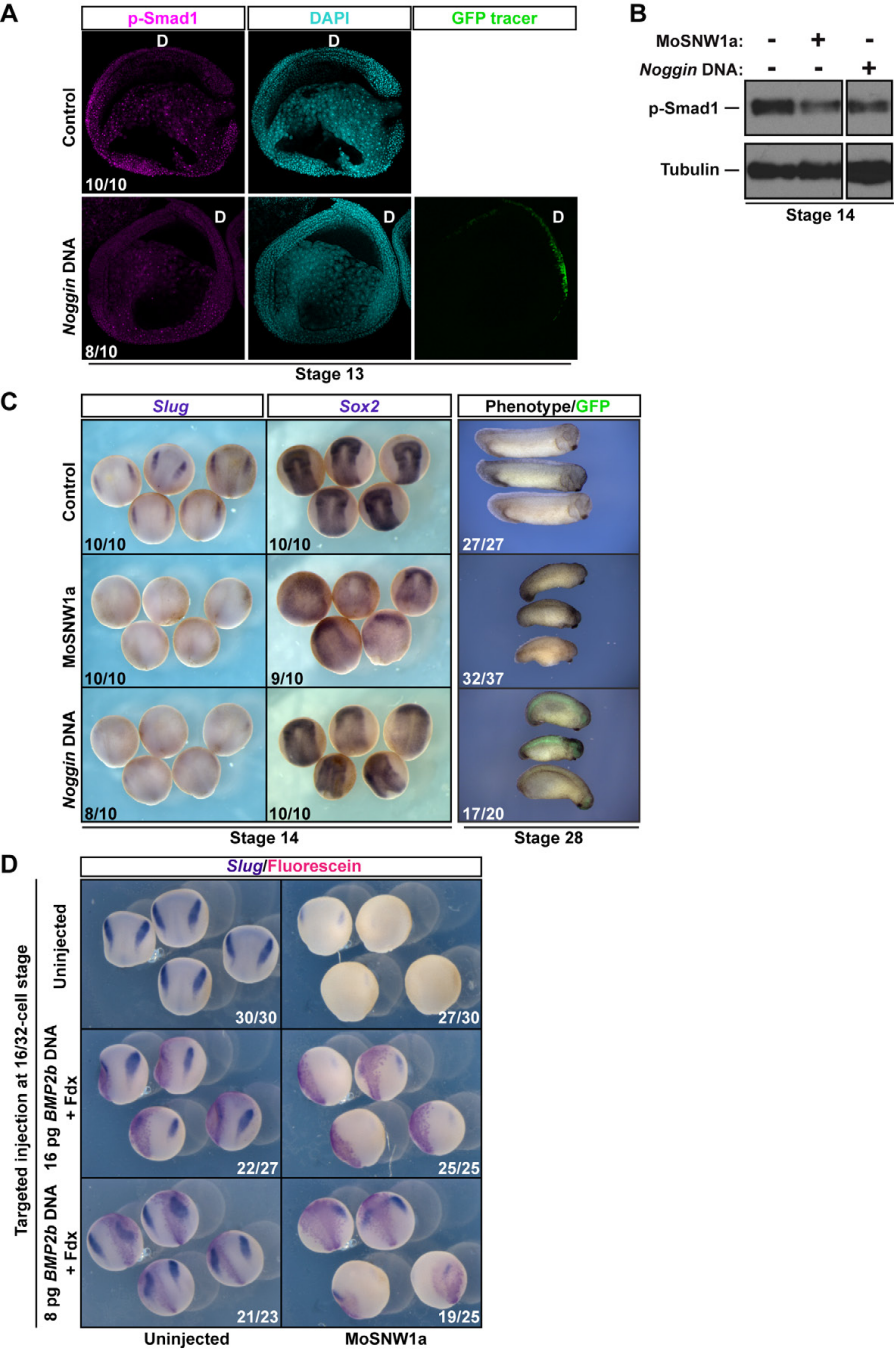
Reduction of BMP signaling by expression of Noggin in the dorsal ectoderm mimics SNW1 depletion, and overexpression of BMP2b rescues the effect of SNW1 depletion. (A–C) Embryos were either uninjected, injected with 20 ng of MoSNW1a at the one-cell stage, or injected with 5 pg of a Noggin expression plasmid and 100 pg of *GFP* mRNA as a tracer in one dorsal A tier blastomere at the 16-cell stage. Samples were fixed at stage 13 for immunostaining for p-Smad1 ([A], pseudo-colored magenta). Samples were taken at stage 14 for Western blot analysis using antibodies against p-Smad1 and Tubulin, the latter serving as a loading control (B), and samples were fixed at stage 14 for WISH using probes against *Slug* and *Sox2* and at stage 28 for phenotype analysis (C). In (A) the nuclei are visualized with DAPI (pseudo-colored cyan) and the Noggin-injected cells with GFP. The dorsal side (D) is marked. In (B) two irrelevant lanes have been removed as indicated by the gap. In the bottom right panel of (C), the GFP marks the region of the embryo expressing exogenous Noggin. (D) *Xenopus* embryos were either uninjected or injected at the one-cell stage with 20 ng of MoSNW1a. At the 16- to 32-cell stage both groups of embryos were injected in one of the A1/B1/A2/B2 blastomeres with either 8 pg of plasmid expressing zebrafish BMP2b or 16 pg of the same plasmid together with Fdx as a tracer, as indicated. Embryos were fixed at stage 14, and neural crest was detected by staining for *Slug* by WISH. The Fdx was visualized with an antibody against fluorescein.

Having shown that the major effect of SNW1 depletion or overexpression is a change in BMP activity, we asked whether this was sufficient to explain the inhibitory effects of SNW1 depletion on neural crest fate and neural plate border formation. We therefore devised an alternative method of specifically lowering BMP activity after the mid-blastula transition to determine whether this would mimic the effect of SNW1 knockdown. We targeted a plasmid expressing the secreted diffusible BMP antagonist Noggin to the dorsal ectoderm, where BMP signaling is normally very low. Noggin produced in this region from mid-blastula transition onwards diffuses into neighboring tissue, thus inhibiting BMP activity. This was sufficient to lower BMP activity in the embryo, equivalent to the effect seen when SNW1 is depleted (Figures 5, 8A, and 8B). Most importantly, we observed a loss of *Slug* staining, disruption of the neural plate border, expansion of the neural plate as judged by *Sox2* staining, and a dorsalized phenotype similar to what we observed with SNW1 depletion (Figure 8C). This strongly supports the idea that BMP activity between stages 12 and 14 is required for neural border sharpening/formation.

### Zebrafish BMP2b Can Rescue the Effect of SNW1 Depletion on Neural Crest Fate in *Xenopus* Embryos

Since we know that zebrafish BMP2b/*Xenopus* BMP4 is responsible for the BMP activity at the neural plate border, we carried out an epistasis experiment to determine whether targeted expression of zebrafish BMP2b post-zygotically on the dorsal side of *Xenopus* embryos could rescue the neural crest phenotype in *SNW1* morphant embryos. We therefore injected a plasmid expressing zebrafish BMP2b into dorsal blastomeres that would give rise to cells in or at the neural plate border. As expected, neural crest cells are very sensitive to the level of BMP activity, and the higher dose attempted (16 pg) resulted in the loss of *Slug* staining on the injected side of control embryos (Figure 8D), suggesting that this dose was too high. Similarly, this dose was unable to rescue neural crest fate in *SNW1* morphant embryos. However, when we decreased the dose by half, we were able to maintain or slightly increase *Slug* staining in control embryos. Very importantly, at this dose, we managed to rescue *Slug* expression on the injected side of a significant number of *SNW1* morphant embryos (Figure 8D). This experiment unequivocally links the effects of SNW1 depletion on BMP activity and the requirement of SNW1 for neural crest fate. Our work has therefore demonstrated that the effect of SNW1 on BMP activity post-gastrulation is critical for neural plate border formation and neural crest fate.

## Discussion

### SNW1 Is a Critical Regulator of BMP Activity in Vertebrate Embryos

We identified SNW1 in a large-scale in vivo functional screen in *Xenopus* for neural crest fate. Our characterization of SNW1 has revealed that it is a key novel regulator of in vivo BMP activity in vertebrate embryos. Specifically, SNW1 is absolutely essential for the regulation of BMP activity at the end of gastrulation and affects critical domains of BMP activity. Using MOs to study the loss-of-function effects of SNW1 in *Xenopus* and zebrafish embryos, we depleted zygotic SNW1, thereby targeting our analysis to post-gastrulation stages of development, and showed that this reduced all readouts of particular domains of BMP activity at these stages of development. Since BMP activity is crucial for dorsal/ventral patterning during gastrulation, it is possible that maternal SNW1 may play important roles in regulating BMP activity and tissue patterning during these earlier developmental stages.

**Figure 9:**
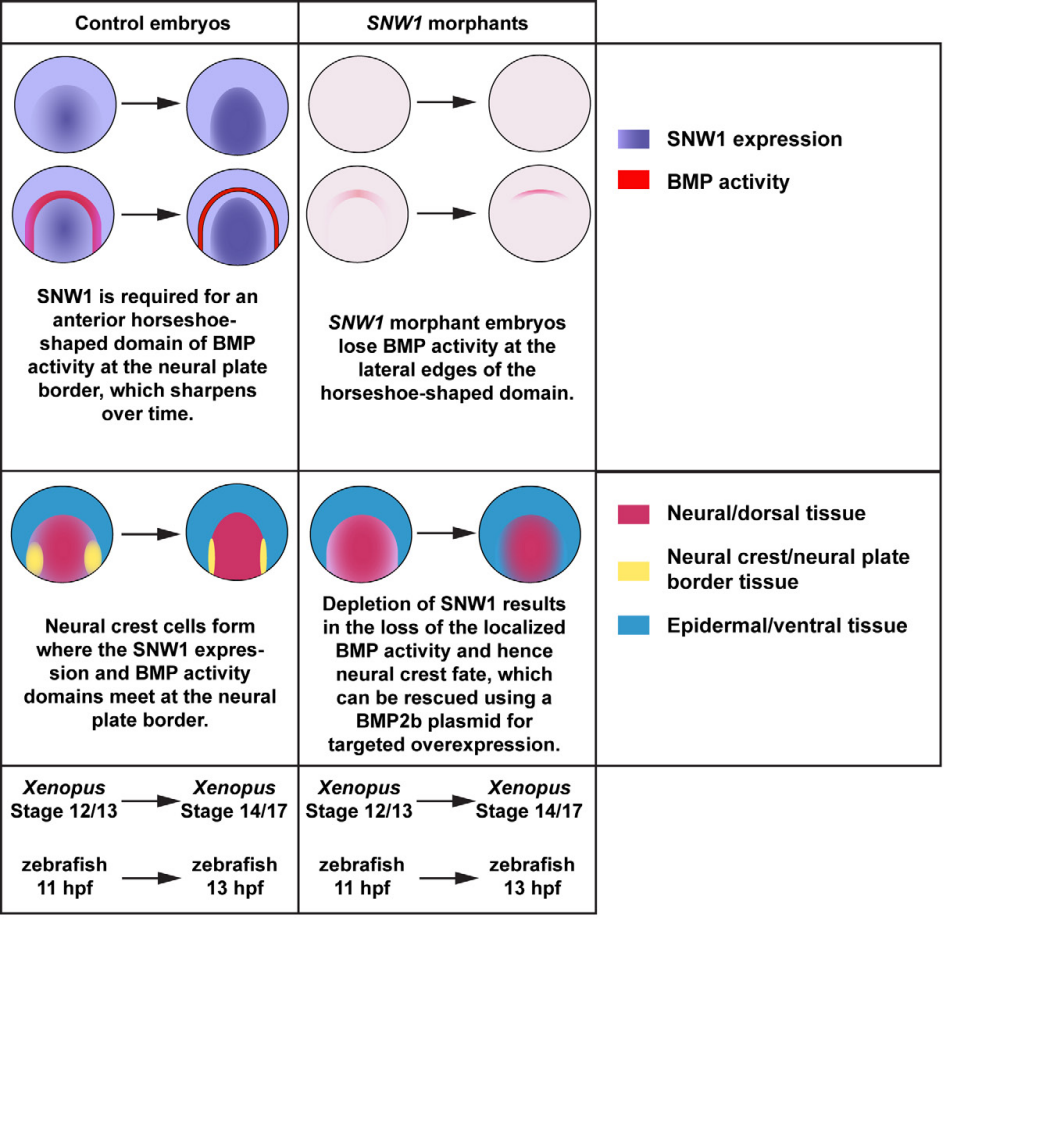
A model of SNW1 function. A schematic diagram summarizing our findings in both *Xenopus* and zebrafish embryos. A dorsal-anterior view of a schematized embryo is shown.

In wild-type embryos, immunostaining for p-Smad1 and analysis of the BRE-*mRFP* fish revealed that BMP activity is spatially and temporally dynamic during the stages of development examined. We have identified a novel horseshoe-shaped domain of BMP activity in 11- to 13-hpf zebrafish embryos at the border between the neural and non-neural ectoderm, and we have evidence that an analogous domain exists in *Xenopus*. Detailed spatial analysis further demonstrated that depletion of SNW1 results in a specific loss of BMP activity in this domain, at the lateral neural plate border (Figure 9), and also on the ventral side of the embryo, but morphant embryos maintain most of the BMP activity found at the tailbud and at the most anterior neural plate.

### SNW1 and BMP Activity on the Dorsal Side of Embryos Are Required for Neural Crest Fate

*SNW1* is enriched dorsally during post-gastrulation stages, and we further isolated the effects of SNW1 depletion on neural crest fate to the dorsal side of the embryo. These results suggest that the BMP activity at the neural plate border, which overlaps with neural crest cells and the enriched domain of *SNW1* expression during the early neurula stage, is responsible for the loss of neural crest fate (Figure 9). Indeed, zygotic expression of the zebrafish BMP2b near or at the neural plate border is sufficient to rescue the loss of neural crest fate in *SNW1* morphant embryos. We did observe additional effects of SNW1 depletion, such as the loss of ventral/posterior tissues in zebrafish (Figure S4B and S4C), and the loss of some ventral BMP activity when SNW1 is depleted in whole embryos may be responsible for this phenotype.

In summary, we have found that disruption of the BMP activity at this particular stage of development (the end of gastrulation) and at a particular location in the embryo (the neural plate border) through the depletion of SNW1 results in the disruption of the sharp neural plate border and virtually complete loss of neural crest fate. This is an ectoderm-specific effect since SNW1 depletion has exactly the same effect when embryos fail to induce mesoderm or undergo gastrulation, as occurs when CerS is overexpressed. We have thus characterized how the BMP activity changes during the neurula stages of development, found a factor that is required for particular domains of BMP activity in the embryo during this time, and demonstrated that this factor plays an essential role in ectodermal patterning.

### SNW1 Is Essential for Neural Plate Border Formation

Consistent with previous reports that the ectoderm responds to signals before or during gastrulation to specify neural tissue [5],[6], we found that *Sox2* and *Sox3* expression is induced in the expected region of the embryo when SNW1 is depleted. However, the expression of these markers is expanded more ventrally, consistent with the dorsalized phenotype of the morphant embryos seen at the tailbud stages. Unlike previous studies where expansion of the neural plate merely shifts the neural plate border, with the neural crest and other border fates still induced [16], we observed a complete loss of border fates. Included in the markers examined, *Noggin* and *Wnt8* are induced in post-gastrulation embryos at the neural plate border region, and this specific domain of expression is lost in *SNW1* morphant embryos (Figure 4A). Since both Noggin and Wnt8 are secreted molecules involved in signaling required for neural crest fate [1],[10], it is possible that this loss of expression contributes to the robust loss of neural crest cells in morphant embryos. However, we think that the loss of their expression at the neural plate border is more likely a consequence of the fact that the border does not form in *SNW1* morphants, because of the loss of BMP signaling in this region, rather than that they are direct targets of SNW1, since expression of *Noggin* and *Wnt8* in the notochord and posterior mesoderm, respectively, is not affected by SNW1 knockdown (Figure 4A).

The two tissues that normally form on either side of the neural crest, neuroectoderm and epidermis, are both specified in *SNW1* morphant embryos, although there is no distinct border between them, as judged by the expression of *Sox2* and *EpiKer,* respectively (Figure 4D). We have demonstrated that SNW1 is a fundamental regulator of BMP activity. To confirm it is SNW1's effects on BMP activity that account for its effects on neural plate border formation at the end of gastrulation, we mimicked the effect of the *SNW1* MO by targeting the zygotic overexpression of a diffusible BMP antagonist, Noggin, to the dorsal ectoderm using DNA injection. Injection of mRNA for overexpression or the non-targeted overexpression of Noggin results in a gross dorsalization of the embryo, with a majority of the ectoderm being respecified as neural (data not shown) [16],[34]. In contrast, localized expression of Noggin after the mid-blastula transition has a milder phenotype, phenocopying the *SNW1* morphant, demonstrating that late alteration of the BMP activity in whole embryos affects just border formation and not neural induction per se.

We also performed the reverse experiment, expression of a BMP ligand via injected exogenous cDNA at the correct stage of development, at the correct locale, and at a correct dose. We showed that this is sufficient to rescue neural crest fate in *SNW1* morphant embryos. If BMP activity however is increased earlier in development through injection of *BMP* mRNA for expression, the embryos develop with a severely ventralized phenotype, with complete loss of neural and head structures (data not shown). Thus, SNW1 is a key regulator of BMP activity at the end of gastrulation, and, at this stage, BMP activity at the neural plate border is absolutely required for the formation of a sharp neural plate boundary and the specification of border fates.

During embryonic development, the same pathways are redeployed at different times and in different contexts to elicit distinct responses. We have demonstrated, to our knowledge for the first time, that neural induction, which is dependent on regulation of BMP activity during gastrulation, is uncoupled temporally from neural border sharpening and border fate specification, which require BMP activity, albeit at a later stage.

### SNW1 Functions Upstream of the BMP Receptors to Regulate BMP Activity

In overexpression studies, SNW1 was previously reported to function with receptor-activated Smad proteins to regulate the transcription of TGF-β superfamily target genes [11],[27], placing it downstream of the receptors. However, we found that SNW1 in a model tissue culture system is not required for either TGF-β- or BMP-dependent transcription, and endogenous SNW1 did not co-immunoprecipitate with any endogenous Smad proteins or Ski. Together, these results argue against SNW1 being a component of the core BMP signal transduction pathway.

Similar to our observations in cell culture experiments, we found that depleting SNW1 in animal caps has absolutely no effect on p-Smad1 levels, whereas intact morphant embryos have substantially lower levels of p-Smad1 compared to control embryos. The animal cap is an isolated, mostly homogeneous tissue, cultured ex vivo. The fact that depletion of SNW1 does not affect p-Smad1 levels in this context further confirms that SNW1 is not a direct component of the BMP pathway downstream of the receptors. Furthermore, we can rule out any effect of SNW1 on the core pathway components specifically in the dorsal ectoderm, as targeted expression of a low dose of *BMP2b* is sufficient to rescue the effects of SNW1 depletion. This indicates that the BMP receptors and downstream signaling pathway must be completely functional in these cells, and SNW1 must thus act upstream.

Since SNW1 is a nuclear factor that has been implicated in transcriptional regulation [11]–[13], we performed a microarray analysis comparing the expression profiles of control versus morphant embryos in an attempt to identify direct targets of SNW1. However, we did not detect any significant changes in the mRNA levels of known modulators, ligands, or components of the BMP signaling pathway (unpublished data). SNW1 has also been reported to be involved in transcriptional elongation and pre-mRNA splicing [14],[15]. We therefore favor the possibility that SNW1 might regulate gene expression, at this level, of components required for BMP receptor activation, which could include the ligands themselves, proteins involved in their activation, extracellular matrix components, or co-receptors.

### The Essential Role of SNW1 in Regulating BMP Activity May Be Evolutionarily Conserved

Since the function of SNW1 is conserved between *Xenopus* and zebrafish and its protein sequence is highly conserved from *C. elegans* to human, its function may also be conserved in other organisms. Indeed, we have evidence that *SNW1*/*Bx42* RNA interference expression in the *Drosophila* wing disc perturbs P-Mad (*Drosophila* phosphorylated Smad1) levels (unpublished data), consistent with reported effects of *SNW1*/*Bx42* RNA interference reducing the expression of Dpp target genes, *Spalt* and *Optomotor Blind* in the same system [35]. In sum, we have identified a novel role for SNW1 in neural plate border sharpening and neural crest fate. Since the neural crest is a vertebrate-specific embryonic tissue type, it is not surprising that this functional effect is a consequence of a more fundamental and likely conserved role that SNW1 plays in regulating BMP activity.

## Materials and Methods

### *Xenopus* and Zebrafish Embryos, Microinjection, and Morpholinos

In vitro fertilization of *X. laevis* embryos and their culture, staging, manipulation, injection, and dissection was carried out as previously described [36], with the exception that animal caps were cultured in Danilchik's blastocoel buffer [37]. Zebrafish were maintained and staged as previously described [38],[39]. Synthetic mRNA for injection was prepared as previously described [36]. *X. tropicalis SNW1* mRNA for injection was synthesized using cDNA template from clone TEgg090l09 from the *X. tropicalis* full-length cDNA library (Source BioScience; [40]), and *X. laevis SNW1* mRNA was synthesized from I.M.A.G.E. Consortium clone ID 5083691. *Noggin* DNA injections were carried out using pCS2-Noggin [34], and *BMP2b* DNA injections used pCS2-zBMP2b. pCS2-zBMP2b was made by amplifying the coding region of BMP2b from zebrafish total gastrula cDNA and cloning the appropriately digested PCR product into pCS2+. The insert was checked by sequencing. *Green fluorescent protein* (*GFP*) mRNA was synthesized from pCS2-GFP (a gift from P. Blader), and *CerS* mRNA from pCS2-CerS [21]. Antisense MOs were designed and obtained from GeneTools and are listed below. In *Xenopus,* MOs were injected at the one- to 32-cell stages as indicated, whilst in zebrafish they were injected at the one- to eight-cell stage.

### Morpholinos

The following MOs were used: MoControl (against human β-globin), CCTCTTACCTCAGTTACAATTTATA; MoSNW1a (against *X. laevis SNW1*), CTAGCGCCATTTTCTCTGTCGATC; MoSNW1b (against *X. laevis SNW1*), GGCTATGGAGGAAGTGACCTAAGAG; and MoZFSNW1 (against zebrafish *SNW1*), ACAGCTTCTCTGCGTCTTACCTTGT).

### The Overexpression Screen

The *X. tropicalis* full-length expressed sequence tag library was obtained from Source BioScience (http://www.lifesciences.sourcebioscience.com/clone-products/cdna-and-orf-resources-/xenopus-tropicalis-est-clones.aspx). At the time of ordering, the collection comprised just over 6,900 unique clones. Pools of 48 individual clones (∼7.5 µg of DNA) were linearized using AscI and used as templates for in vitro transcription reactions with SP6 RNA polymerase to generate 5′ capped mRNA for injection into one-cell *X. laevis* embryos. Embryos were fixed at stage 14 and stained for *Slug* expression. Pools showing a strong effect on *Slug* staining were then deconvoluted. Individual clones isolated after deconvolution include the following: *Dkk-1* (TGas131c10), c*-Myc* (TEgg042l13), *Noggin2* (TNeu122a14), and *SNW1* (TEgg090l09).

### Cloning of Zebrafish *SNW1*

Zebrafish *SNW1* cDNA including 5′ UTR was amplified from total cDNA (somitogenesis stage) using the following primers: 5′-TCCAAGATGTCGCTTACAAG-3′ and 5′-CAGAGAAAGTGTCTCACTCC-3′. The PCR product was cloned into pGEM-T (Promega) and sequence verified.

### Generation of the Transgenic BRE-*mRFP* Zebrafish Line

A detailed report of the transgenic BRE-*mRFP* line will be published elsewhere. Briefly, the construct used to generate pGL3-BRE-mRFP was the pGL3-BRE-Luciferase plasmid [41], in which we replaced the *luciferase* gene with *mRFP*. The BRE-mRFP sequence was further subcloned into the TOL2 vector to generate a stable transgenic line as described [42]–[44]. An *mRFP* antisense in situ probe was generated by cloning a full-length *mRFP* cDNA into pGEM-T (Promega).

### WISH, Double In Situ Hybridization, and Immunostaining

WISH was performed in *Xenopus* and zebrafish essentially as previously described [45],[46]. The probes are described below. The chromogenic reaction was carried out using BM purple alkaline phosphatase substrate (Roche) or NBT/BCIP (Sigma). Fdx (Molecular Probes) was visualized with an anti-fluorescein antibody (11426338910; Roche). The double in situ protocol in zebrafish was modified from [47] using digoxigenin- and fluorescein-labeled antisense probes. The chromogenic substrates used were NBT/BCIP (Sigma) in combination with BCIP (Roche) or INT/BCIP (Roche). The use of BCIP as the first chromogenic substrate results in turquoise staining. However, when used after NBT/BCIP, BCIP gives rise to grey-purple staining. INT/BCIP was used as the second chromogenic substrate for *Hgg1* detection. For double in situ hybridization in *Xenopus*, embryos were incubated with two differentially labeled probes during hybridization. The first probe was developed as described for single in situ hybridizations. For the second probe, embryos were washed three times in methanol, then rehydrated in decreasing concentrations of ethanol until 100% PBS-Tween was reached, before repeating the antibody incubation and staining steps. The second chromogenic substrate used was either BCIP alone or Magenta Phos (Sigma). Immunostaining of *Xenopus* embryos was carried out as previously described [48] using a 1∶200 dilution of p-Smad1 antibody (9511; Cell Signaling Technology). Immunostaining of zebrafish embryos was as previously described [4].

### WISH Probes

Digoxigenin-UTP (Roche)–labeled antisense RNA probes were synthesized using cDNA templates encoding *Snail* [49], *Slug* [16], *Sox9* [17], *Twist* [18], *FoxD3* [50], *Sox2* [16], *Sox3* [51], *Wnt8* [52], *Noggin* [34], *Xbra* [53], *Xnot* [54], *Chordin* [55], *Goosecoid* [56], *BMP4* [57], *Sizzled* [58], *Zic3* [59], *Otx2* [60], *MyoD* [61], *Epidermal keratin* [62], *Sox10* [63], *Foxd3* (zebrafish; [64]), *MyoD* (zebrafish; [65]), *N-cadherin* [66], *Otx2* (zebrafish; [67]), *Cdx4* [68], *Even-skipped-like 1* [69], *No tail a* [70], *BMP2b* [71], *BMP7* [72], and *Hgg1* [73]. The antisense probe against *Msx1* was generated from the plasmid XMsx1 in pGEM4Z and protects the region of *Msx1* encoding amino acids 40–129. The antisense probe against zebrafish *BMP4* was generated from the plasmid pCS2P+-zBMP4 (a gift from Arne Lekven) and protects full-length *BMP4*. The probe against *Xenopus SNW1* was generated from pGEM-T-XlSNW1 and protected the region of *SNW1* encoding amino acids 102–300. The probe against zebrafish *SNW1* was generated from pGEM-T-zSNW1 and protects full-length *SNW1*.

### Alcian Blue Staining

Alcian blue staining of cartilage was carried out as previously described [8].

### Western Blotting

*Xenopus* embryos or animal caps were snap frozen at the required stage, and extracts were prepared as previously described [74]. Mammalian cell extracts were prepared as described in [31]. For zebrafish protein extracts, typically ten embryos (including chorion) were snap frozen and subsequently processed using the same protocol as for *Xenopus* embryos, except that the embryos were lysed using a plastic pestle and a pellet pestle motor (Kontes). Western blotting was performed using standard techniques and the following antibodies: from Santa Cruz Biotechnology, anti-SNW1 (H-300), anti-Smad4 (B8), anti-MCM6 (sc-30139), anti-Ski (H-329); from Abcam, anti-SNW1 (ab67715; for Figure 7A), anti-Tubulin (ab6160); from Cell Signaling Technology, anti-p-Smad1 (9511); from Millipore, anti-p-Smad2 (clone A5S); from Zymed, anti-Smad1 (38-5400); and from BD Biosciences, anti-Smad2/3. Each lane on the Western blots is an average of 10–15 embryos, with 1–1.5 embryo equivalents of extract loaded.

### RNA Extraction from *Xenopus* Embryos, RT-PCR, and qPCR

Extraction of total RNA from *Xenopus* embryos was carried out using Trizol reagent (Invitrogen) followed by a cleanup and on-column DNase treatment using the RNeasy Mini Kit (Qiagen). The AffinityScript QPCR cDNA Synthesis Kit (Stratagene) was used for cDNA synthesis following the manufacturer's protocol. Semi-quantitative PCR from cDNA templates was carried out using GoTaq (Promega) on a GeneAmp PCR system 9700 (Applied Biosystems), and qPCR was performed using an ABI 7500 Fast system (Applied Biosystems) with SYBR Green Master Mix (Applied Biosystems). The primers were as follows: *SNW1*, 5′-TGATGCTATTGCTCGACAGG-3′ and 5′-CTTCTGGGACACGGATTTGT-3′; *Chordin*, 5′-CATGCTCTTTCGAAGGTCAA-3′ and 5′-GATCACAAATCACGGTACGC-3′ [75]; *Sizzled*, 5′-TGCCGTAGTATGTGTGTAGCTG-3′ and 5′-ACTCTTTGCTGAGAGTGTCCAA-3′ [75]; *Slug*, 5′-CACGTTACCCTGCGTCTGTA-3′ and 5′-GCAGGTGGGCTCTTAAGTTG-3′; and *ODC*, 5′-ACAAAGAAACCCAAACCAGA-3′ and 5′-CAAACAACATCCAGTCTCCAA-3′ [75].

### MDA-MB-231 Cell Culture and siRNA Transfection

MDA-MB-231, MDA-MB-231 CAGA_12_-Luc/TK-Renilla and MDA-MB-231 BRE-Luc/TK-Renilla cell lines were cultured in DMEM supplemented with 10% FBS. siRNA transfection was carried out using INTERFERin siRNA transfection reagent (PolyPlus Transfection) with 1 nM of siRNA. Cells were incubated after transfection for 72 h before further processing. siRNAs were purchased from Dharmacon. RISC-Free siControl (D-001220-01-05); Human Smad4 SMARTpool (M-003902-01); Human SNW1 SMARTpool (L-012446-00); Human SNW1-5 (J-012446-05); Human SNW1-6 (J-012446-06); Human SNW1-7 (J-012446-07); Human SNW1-8 (J-012446-08); and ON-TARGETplus Control Non-targeting siRNA 1 (D-001810-01).

### Luciferase Assays and Co-Immunoprecipitations

MDA-MB-231 cells stably expressing Renilla from the Herpes Simplex Virus Thymidine Kinase (HSVtk) promoter (Promega) and Luciferase from either the TGF-β-dependent promoter CAGA_12_ [76] or the BMP-dependent promoter BRE [41] were used to study signal-induced transcription. Luciferase and Renilla were assayed as previously described [31]. Co-immunoprecipitations (IPs) were carried out as previously described [31].

### In Vitro Translation

The T_N_T Coupled Reticulocyte Lysate System (Promega) was used for direct translation of in vitro transcribed mRNA, and translation was carried out according to the manufacturer's protocol. ^35^S-labeled methionine and unlabeled amino acids were added to a 20 µl of reticulocyte lysate reaction along with 4 µg of mRNA template (same mRNA:volume ratio as injected into embryos). For reactions where MOs were added to block translation, 400 ng of MO was added (same MO:volume ratio as MOs injected into embryos). Samples were separated on a 15% SDS gel, which was Coomassie stained before being dried and exposed to Hyperfilm (Amersham) to detect protein bands positive for ^35^S-Met.

## Supporting Information

Figure S1 **SNW1 is a highly conserved protein.** Multiple sequence alignment of SNW1 protein sequences from various model organisms using ClustalW. The *X. laevis* SNW1 protein (NP_001089903) is 98% identical to the *X. tropicalis* SNW1 (NP_001017145), 88% identical to *Homo sapiens* SNW1 (NP_036377), 87% identical to *Mus musculus* SNW1 (NP_079783), 87% identical to *Gallus gallus* SNW1 (XP_421294), 83% identical to the *Danio rerio* SNW1 (AAI07987), 62% identical to the *Drosophila melanogaster* ortholog Bx42 (NP_511093), 53% identical to the *C. elegans* ortholog SKP-1 (NP_505950), and only 22% identical to *Saccharomyces cerevisiae* ortholog PRP45 (NP_009370). SNW1 is named based on the completely conserved amino acid signature found between amino acids 252–256 in the *X. laevis* protein (outined in red), which is central to the SNW domain (outlined in orange). In some organisms SNW1 is also known as SKIP (Ski-interacting protein) [32]. In all organisms except yeast, SNW1 has a C-terminal nuclear localization signal (NLS; outlined in green), suggesting that it is a nuclear protein.
(2.41 MB TIF)

Figure S2 **SNW1 is ubiquitously expressed in the** ***Xenopus*** **embryo.** (A) Total RNA was extracted from eggs (E) or embryos at different stages as indicated and analyzed by semi-quantitative RT-PCR for *SNW1* and *Slug*, with *Ornithine decarboxylase* (*ODC*), which is ubiquitously expressed at a constant level [77] as a loading control. The neural-crest-specific marker *Slug* is a positive control for a gene induced at neural stages. To demonstrate specificity of the products, the reactions were performed with (+RT) or without (-RT) reverse transcriptase. (B) Total RNA was extracted from whole embryos or the dorsal or ventral halves of bisected embryos at the stages indicated and analyzed by qPCR for *SNW1*, and the dorsally and ventrally expressed controls *Chordin* (*Chd*) and *Sizzled* (*Szl*), respectively.
(0.15 MB TIF)

Figure S3 **Depletion of SNW1 results in a dorsalized phenoype, reduces the number of** ***Snail*** **-positive neural crest cells, disrupts migration of neural crest cells into the branchial arches at the tailbud stages, and results in loss of cranial cartilage.** (A) MOs used to deplete SNW1 in *Xenopus* and zebrafish. The two translation-blocking MOs targeting *X. laevis* (*Xl*) *SNW1* are shown and aligned with both the *X. laevis* and *X. tropicalis* (*Xt*) sequences for *SNW1*. MoSNW1a overlaps the ATG (highlighted in green), while MoSNW1b binds in the 5′ UTR. The MOs are 100% complementary to the *X. laevis SNW1* sequence, whereas there are five non-consecutive mismatches against the *X. tropicalis* sequence for each MO. The sequence targeted by the zebrafish *SNW1* splice-blocking MO is shown. The exon is shown in uppercase and the intron in lowercase. (B) In vitro translation using rabbit reticulocyte lysate of either *X. laevis* or *X. tropicalis SNW1* in the absence or presence of MoSNW1a or MoSNW1b. Translation of *X. laevis SNW1* is inhibited by both MOs, with MoSNW1a being the more efficient. Neither MO inhibits translation of *X. tropicalis SNW1*. SNW1 levels were detected by labeling with ^35^S-methionine and autoradiography. Equal amounts of translation reaction were loaded in each lane. (C) One-cell embryos were injected with 40 ng of control MO (C), 20 ng of MoSNW1a (Ma), or 40 ng of MoSNW1b (Mb). Whole embryo extracts were prepared at the stages indicated and analyzed by Western blotting for SNW1, and Tubulin as a loading control. Both MOs are effective in blocking zygotic SNW1 translation, as evidenced by a reduction in SNW1 protein levels only from stage 12 onwards, with Ma being more efficient than Mb, consistent with (B). (D) One-cell embryos were injected as in (C). Embryos were fixed at stage 28 and analyzed by WISH for *Snail*, which reveals that cranial neural crest induction is disrupted. (E) Injection of MoSNW1a in one cell of a two-cell embryo reduces cranial cartilage formation on the injected side (arrows). Two-cell *Xenopus* embryos were injected in one cell with either 20 ng of control MO or 20 ng of MoSNW1a, along with 250 pg of *GFP* mRNA as a tracer. In (D) and (E) the number of embryos out of the total analyzed that showed the presented phenotype is given.
(2.74 MB DOC)

Figure S4 **Overexpression of SNW1 can rescue the effects of SNW1 depletion in** ***Xenopus*** **and zebrafish.** (A) The effects of SNW1 knockdown in *X. laevis* embryos can be rescued by overexpression of *X. tropicalis SNW1*. Embryos injected with 20 ng of control MO, 20 ng of MoSNW1a, 500 pg of *X. tropicalis SNW1* mRNA, or both MoSNW1a and *X. tropicalis SNW1* mRNA at the one-cell stage. Neural crest induction was assayed by WISH for *Slug* and *Sox9* at stage 16. The phenotype was analyzed at stage 30. The number of embryos out of the total analyzed that showed the presented staining pattern/phenotype is given. (B) Zebrafish embryos were injected with 15 ng of either control MO or MoZFSNW1. They were photographed for phenotype analysis at 28 hpf. The morphant embryos present a dorsalized-like phenotype, but also display necrosis in the head, which is not rescued by p53 MO co-injection (data not shown) [78]. (C) Overexpression of *X. laevis SNW1* partially rescues the effects of SNW1 knockdown in zebrafish. Embryos were either uninjected or injected with 7.5 ng of MoZFSNW1, 125 pg of *X. laevis SNW1* mRNA, or both. Embryos were cultured until 40 hpf, when they were analyzed for phenotype. MO-injected embryos and embryos injected with both the MO and the rescue mRNA were scored for a dorsalized phenotype (looking only at the extent of posterior structures) as in [71] (righthand graph).
(5.35 MB DOC)

Figure S5 **SNW1 knockdown has no effect on mesoderm induction or gastrulation and does not affect Activin-dependent induction of mesodermal tissue in** ***Xenopus*** **animal caps.** (A) One-cell *Xenopus* embryos were injected with 20 ng of MoSNW1a. Uninjected control and MoSNW1a-injected embryos were fixed at stage 12 for WISH using probes against *Xbra*, *Goosecoid*, and *Sizzled*. (B) Animal caps dissected from MoSNW1a-injected embryos elongate similarly in response to 20 ng/ml Activin (PeproTech) as caps cut from control embryos. Thus mesoderm induction is not affected by injection of MoSNW1a. (C) Zebrafish embryos were injected with 15 ng of either control MO or MoZFSNW1. The embryos were fixed at 12 hpf and stained for the mesoderm markers *No tail a* (axial and tail), *Even-skipped-like 1* (paraxial and tail), and *Cdx4* (posterior axial and tail as well as some ectoderm). In *SNW1* morphants, the expression of the mesoderm markers is largely unchanged, but there is a slight reduction in their expression in the tail mesoderm. *Otx2* is a marker for anterior neural ectoderm and is normal albeit slightly expanded in *SNW1* morphants. *Otx2* and *Cdx4* expression also indicate that anterior/posterior patterning is preserved in the absence of SNW1. In all cases the number of embryos out of the total analyzed that showed the presented staining pattern is given.
(3.44 MB TIF)

Figure S6 **The BRE-** ***mRFP*** **transgenic zebrafish line is a reporter for BMP activity.** (A) 24-h transgenic embryos imaged using bright field or fluorescence microscopy, or processed for in situ hybridization using a probe against *mRFP*. mRFP protein or mRNA is indicative of BMP activity. Indeed, in the anterior/head region, mRFP is detected in known sites of BMP expression and/or activity such as the dorsal retina and lens (arrow; [79]) and the otic placode (arrowhead; [80]). mRFP is also strongly detected in the epiphysis/pineal gland, consistent with data obtained in *Xenopus* (asterisk; [81]). In the posterior/tail region, mRFP as a readout of BMP signaling is present for instance in the dorsal ectoderm, where BMP ligands are expressed (arrow; [82]), the cloaca (arrowhead; [83]), and the somites (S; [82]). (B) *mRFP* in situ hybridization on transgenic embryos at 85% epiboly. *mRFP* transcripts are detected in the ventral ectoderm and the ventral lateral plate and intermediate mesoderm, where BMP signaling is known to be active [26]. The *mRFP* pattern is consistent with p-Smad1/5 staining at the same stage (arrowheads; see also [4]). (C) Noggin overexpression inhibits *mRFP* transcription downstream of the BRE promoter. As expected, injection of *X. laevis Noggin* mRNA results in reduced BMP signaling [26], which leads to lower amounts of *mRFP* transcripts in 70% epiboly embryos. Arrows and arrowhead indicate the extent of the *mRFP* expression domain for comparison between uninjected and injected embryos. In (B) and (C), V, ventral; D, dorsal. The number of embryos out of the total analyzed that showed the presented staining pattern is given.
(2.69 MB TIF)

Figure S7 **BMP2b is the BMP family member in zebrafish that accounts for the BMP activity at the epidermis/neural ectoderm border that is dependent on SNW1.** (A) Comparison of *BMP2b, BMP4,* and *BMP7* expression in zebrafish embryos at 11 hpf (2–3 somite stage) using WISH. *BMP2b* transcripts are enriched at the epidermis/neural ectoderm border (arrows). In contrast, *BMP4* is enriched in the anterior prechordal plate and the tailbud, while *BMP7* appears to be expressed in the endoderm. (B) Transgenic BRE-*mRFP* embryos were injected with 15 ng of control MO or MoZFSNW1. They were fixed at 11 hpf, when WISH was performed for *mRFP*. SNW1 knockdown results in the strong loss of BMP activity at the epidermis/neural ectoderm border, as seen with *mRFP* WISH in BRE-*mRFP* embryos. Notably, some of the posterior *mRFP* staining is preserved in the *SNW1* morphant, which is likely because of BMP4 activity. *BMP2b* expression, however, appears unaltered in *SNW1* morphants, suggesting that SNW1 does not induce BMP activity at the epidermis/neural ectoderm border through the transcriptional regulation of *BMP2b*. Note that the same batch of injected embryos was stained for *mRFP* or *BMP2b*.
(2.22 MB TIF)

Figure S8 **Localized BMP activity is detected at the neural plate border region in** ***Xenopus*** **embryos, and this is dependent on SNW1.** Fdx with or without 20 ng of MoSNW1a was injected into one cell of two-cell embryos. They were fixed at stage 13, bisected transversely through the neural crest region, and immunostained with antibodies against p-Smad1 and fluorescein. Nuclei were stained with DAPI. The specific p-Smad1 staining is nuclear and punctate. White arrowheads indicate a domain of p-Smad1 staining in the neural crest region that is lost on the injected side when SNW1 is depleted with the MO.
(2.76 MB TIF)

Figure S9 **siRNA-mediated knockdown of SNW1 in mammalian cells does not affect TGF-β signaling.** (A) siRNA-mediated knockdown of SNW1 in mammalian cells does not affect a TGF-β-responsive reporter activity. An MDA-MB-231 cell line stably expressing TK-Renilla and CAGA_12_-Luciferase was transfected with a non-targeting siRNA (NT), an siRNA pool against *SNW1*, the four separate siRNAs that make up the pool, or an siRNA against *Smad4*. 72 h after transfection, cells were induced with 2 ng/ml TGF-β as indicated. Samples were taken for Western blotting after 1 h and for Luciferase/Renilla assays after 8 h. Whereas knockdown of Smad4 diminishes the CAGA_12_-Luciferase reporter activity, SNW1 depletion does not significantly alter TGF-β-dependent transcription. The efficiency of knockdown is demonstrated in the Western blots shown. (B) SNW1 does not interact with Ski or Smad proteins at endogenous levels in cells. MDA-MB-231 cells were preincubated for 3 h with 50 µM MG132 (Sigma) to prevent Ski degradation in response to TGF-β, before being induced or not with 2 ng/ml TGF-β for 1 h. Cell were then lysed in 150 mM co-IP buffer and immunoprecipitated with the antibodies indicated. A “beads alone” IP served as a negative control (lanes 3 and 4), and 10% of the total lysate used per IP was reserved for input (lanes 1 and 2). The IPs were blotted with the antibodies indicated. Smad2/3 and p-Smad2 as well as Ski can be co-immunoprecipitated with Smad4, as expected [28], while SNW1 was not co-immunoprecipitated (lanes 5 and 6). Immunoprecipitated SNW1 did not co-immunoprecipitate Smad2/3, Smad4, or Ski (lanes 7 and 8). Only Smad2/3, p-Smad2, and Smad4 co-immunoprecipitated with Ski (lanes 9 and 10). The blot for Smad2/3 is a re-probe of the SNW1 blot, and thus the SNW1 signal can additionally be seen on the Smad2/3 blot.
(0.46 MB TIF)

## Abbreviations

BMP: bone morphogenetic protein
BRE: BMP responsive element
CerS: Cerberus short
Fdx: fluorescein dextran
GFP: green fluorescent protein
hpf: hours post-fertilization
IP: immunoprecipitation
MO: morpholino
mRFP: monomeric red fluorescent protein
p-Smad1: phosphorylated Smad1
qPCR: quantitative PCR
RT-PCR: reverse transcription PCR
siRNA: small interfering RNA
TGF-β: transforming growth factor β
WISH: whole-mount in situ hybridization
