# Supplementary figures and images for: SNW1 Is a Critical Regulator of Spatial BMP Activity, Neural Plate Border Formation, and Neural Crest Specification in Vertebrate Embryos
Source: PLoS Biol. 2011 Feb 15;9(2):e1000593. doi: 10.1371/journal.pbio.1000593 (PMC3039673; doi:10.1371/journal.pbio.1000593)

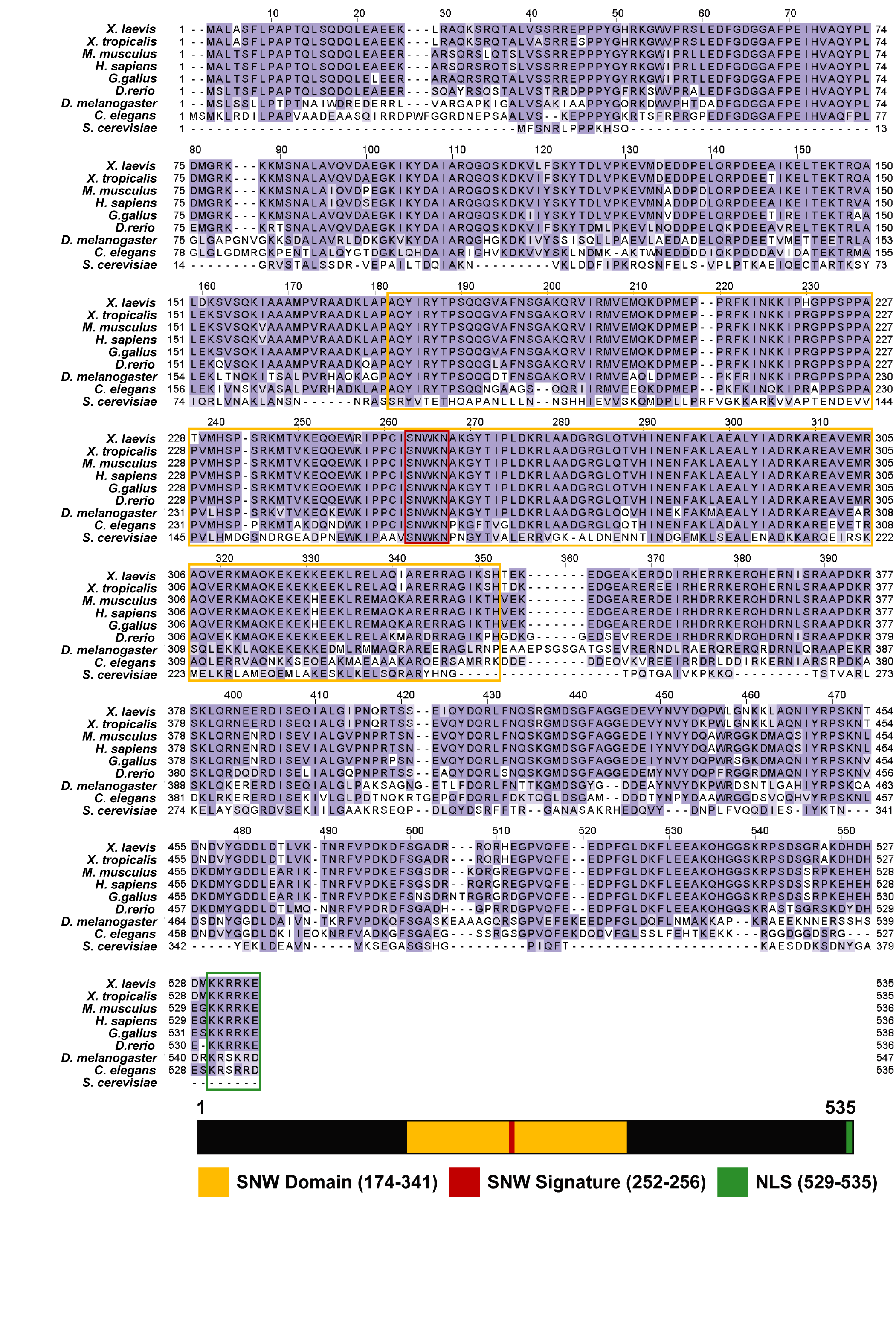

Supplement: Figure S1 — SNW1 is a highly conserved protein. Multiple sequence alignment of SNW1 protein sequences from various model organisms using ClustalW. The X. laevis SNW1 protein (NP_001089903) is 98% identical to the X. tropicalis SNW1 (NP_001017145), 88% identical to Homo sapiens SNW1 (NP_036377), 87% identical to Mus musculus SNW1 (NP_079783), 87% identical to Gallus gallus SNW1 (XP_421294), 83% identical to the Danio rerio SNW1 (AAI07987), 62% identical to the Drosophila melanogaster ortholog Bx42 (NP_511093), 53% identical to the C. elegans ortholog SKP-1 (NP_505950), and only 22% identical to Saccharomyces cerevisiae ortholog PRP45 (NP_009370). SNW1 is named based on the completely conserved amino acid signature found between amino acids 252–256 in the X. laevis protein (outined in red), which is central to the SNW domain (outlined in orange). In some organisms SNW1 is also known as SKIP (Ski-interacting protein) [32]. In all organisms except yeast, SNW1 has a C-terminal nuclear localization signal (NLS; outlined in green), suggesting that it is a nuclear protein. (2.41 MB TIF) [file pbio.1000593.s001.tif]

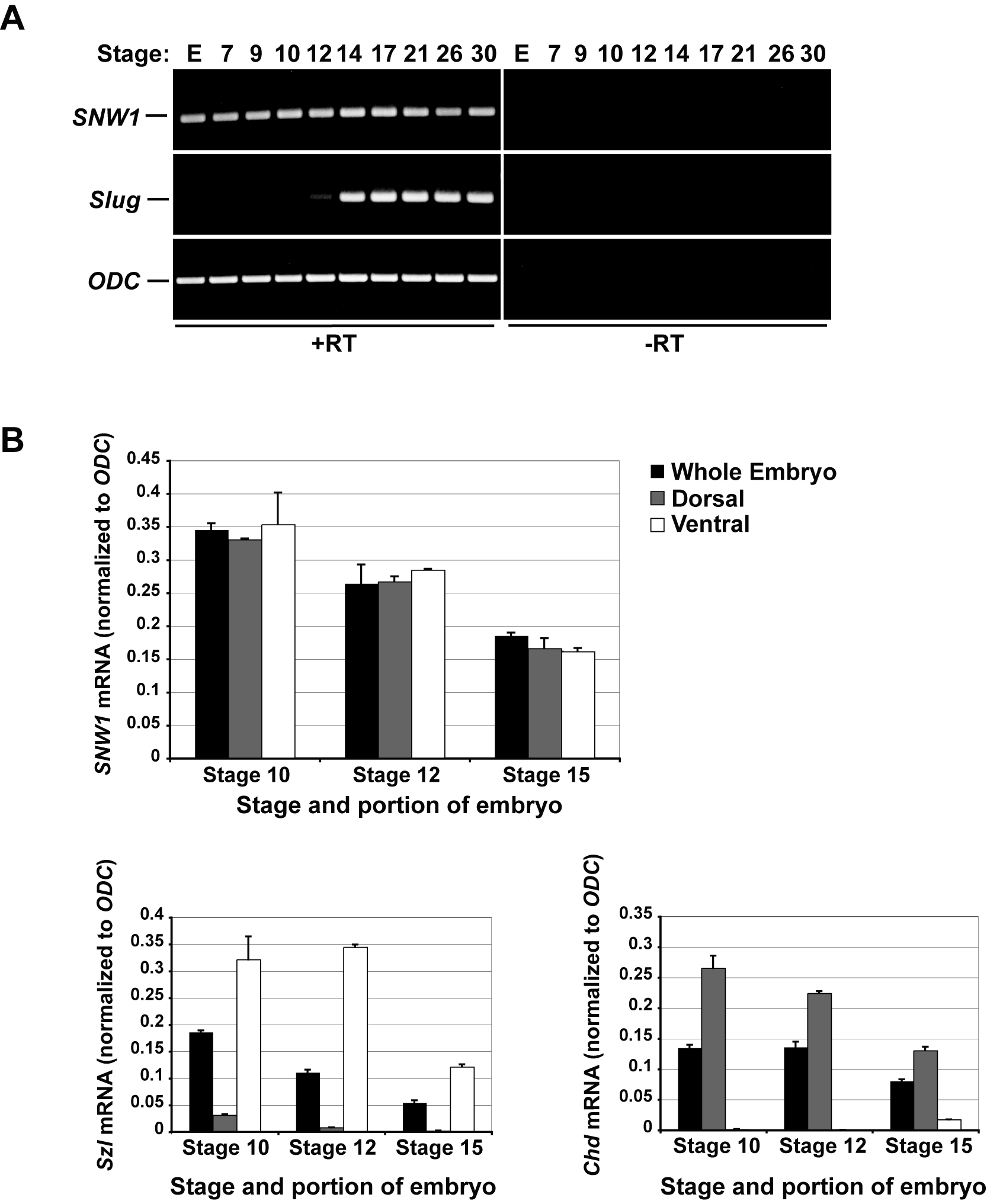

Supplement: Figure S2 — SNW1 is ubiquitously expressed in the Xenopus embryo. (A) Total RNA was extracted from eggs (E) or embryos at different stages as indicated and analyzed by semi-quantitative RT-PCR for SNW1 and Slug, with Ornithine decarboxylase (ODC), which is ubiquitously expressed at a constant level [77] as a loading control. The neural-crest-specific marker Slug is a positive control for a gene induced at neural stages. To demonstrate specificity of the products, the reactions were performed with (+RT) or without (-RT) reverse transcriptase. (B) Total RNA was extracted from whole embryos or the dorsal or ventral halves of bisected embryos at the stages indicated and analyzed by qPCR for SNW1, and the dorsally and ventrally expressed controls Chordin (Chd) and Sizzled (Szl), respectively. (0.15 MB TIF) [file pbio.1000593.s002.tif]

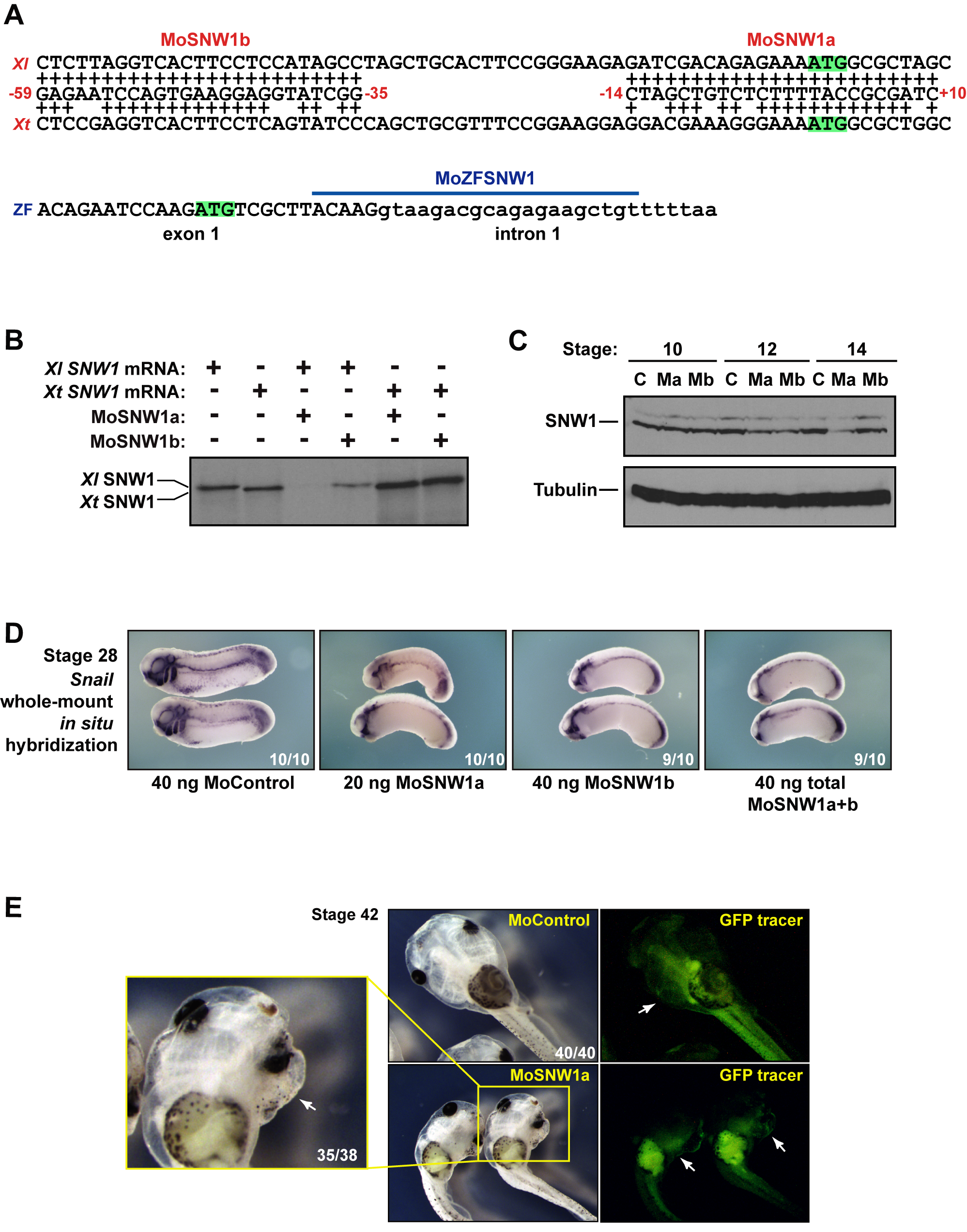

Supplement: Figure S3 — Depletion of SNW1 results in a dorsalized phenoype, reduces the number of Snail -positive neural crest cells, disrupts migration of neural crest cells into the branchial arches at the tailbud stages, and results in loss of cranial cartilage. (A) MOs used to deplete SNW1 in Xenopus and zebrafish. The two translation-blocking MOs targeting X. laevis (Xl) SNW1 are shown and aligned with both the X. laevis and X. tropicalis (Xt) sequences for SNW1. MoSNW1a overlaps the ATG (highlighted in green), while MoSNW1b binds in the 5′ UTR. The MOs are 100% complementary to the X. laevis SNW1 sequence, whereas there are five non-consecutive mismatches against the X. tropicalis sequence for each MO. The sequence targeted by the zebrafish SNW1 splice-blocking MO is shown. The exon is shown in uppercase and the intron in lowercase. (B) In vitro translation using rabbit reticulocyte lysate of either X. laevis or X. tropicalis SNW1 in the absence or presence of MoSNW1a or MoSNW1b. Translation of X. laevis SNW1 is inhibited by both MOs, with MoSNW1a being the more efficient. Neither MO inhibits translation of X. tropicalis SNW1. SNW1 levels were detected by labeling with 35S-methionine and autoradiography. Equal amounts of translation reaction were loaded in each lane. (C) One-cell embryos were injected with 40 ng of control MO (C), 20 ng of MoSNW1a (Ma), or 40 ng of MoSNW1b (Mb). Whole embryo extracts were prepared at the stages indicated and analyzed by Western blotting for SNW1, and Tubulin as a loading control. Both MOs are effective in blocking zygotic SNW1 translation, as evidenced by a reduction in SNW1 protein levels only from stage 12 onwards, with Ma being more efficient than Mb, consistent with (B). (D) One-cell embryos were injected as in (C). Embryos were fixed at stage 28 and analyzed by WISH for Snail, which reveals that cranial neural crest induction is disrupted. (E) Injection of MoSNW1a in one cell of a two-cell embryo reduces cranial cartilage formation on the inject [file pbio.1000593.s003.tif]

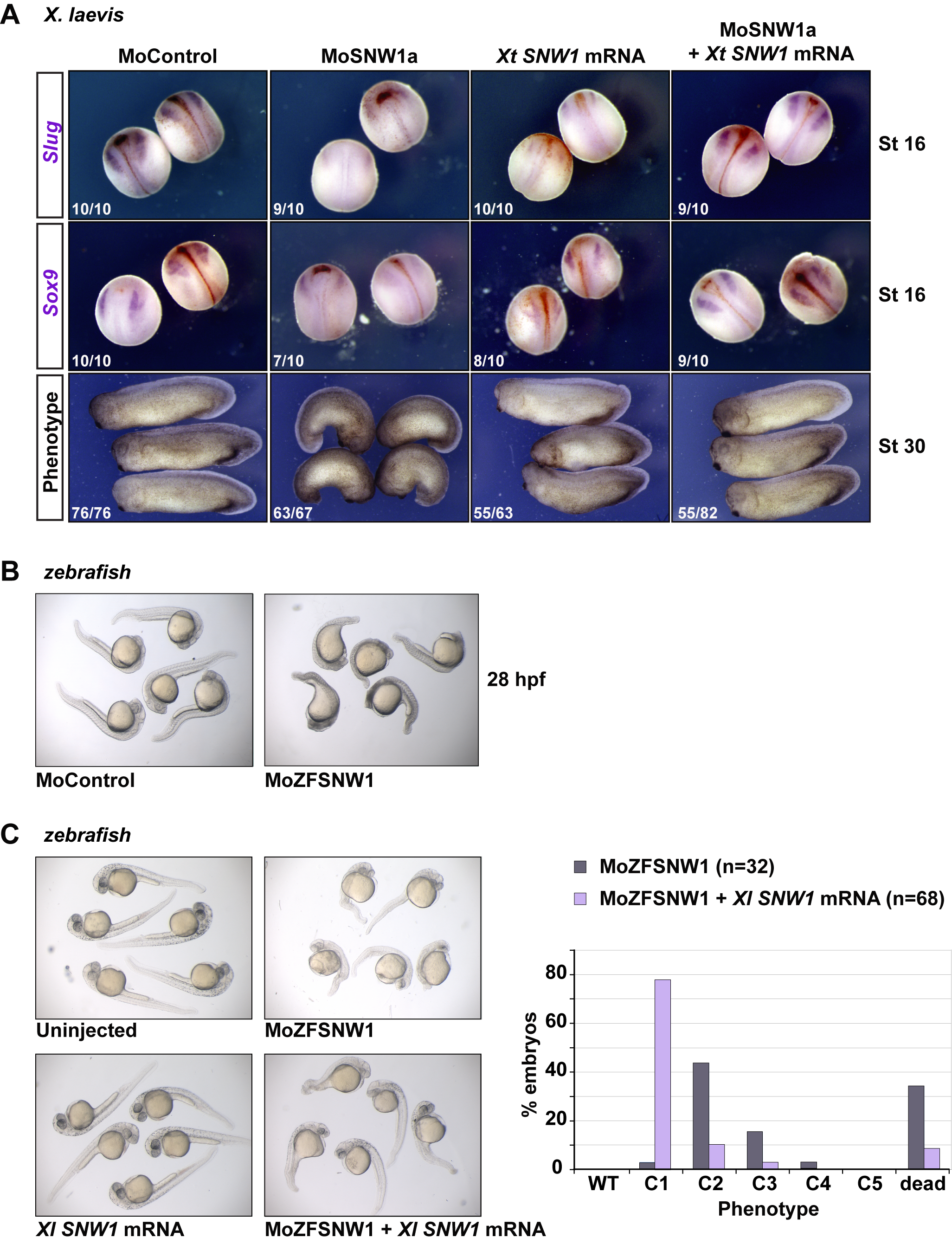

Supplement: Figure S4 — Overexpression of SNW1 can rescue the effects of SNW1 depletion in Xenopus and zebrafish. (A) The effects of SNW1 knockdown in X. laevis embryos can be rescued by overexpression of X. tropicalis SNW1. Embryos injected with 20 ng of control MO, 20 ng of MoSNW1a, 500 pg of X. tropicalis SNW1 mRNA, or both MoSNW1a and X. tropicalis SNW1 mRNA at the one-cell stage. Neural crest induction was assayed by WISH for Slug and Sox9 at stage 16. The phenotype was analyzed at stage 30. The number of embryos out of the total analyzed that showed the presented staining pattern/phenotype is given. (B) Zebrafish embryos were injected with 15 ng of either control MO or MoZFSNW1. They were photographed for phenotype analysis at 28 hpf. The morphant embryos present a dorsalized-like phenotype, but also display necrosis in the head, which is not rescued by p53 MO co-injection (data not shown) [78]. (C) Overexpression of X. laevis SNW1 partially rescues the effects of SNW1 knockdown in zebrafish. Embryos were either uninjected or injected with 7.5 ng of MoZFSNW1, 125 pg of X. laevis SNW1 mRNA, or both. Embryos were cultured until 40 hpf, when they were analyzed for phenotype. MO-injected embryos and embryos injected with both the MO and the rescue mRNA were scored for a dorsalized phenotype (looking only at the extent of posterior structures) as in [71] (righthand graph). (5.35 MB DOC) [file pbio.1000593.s004.tif]

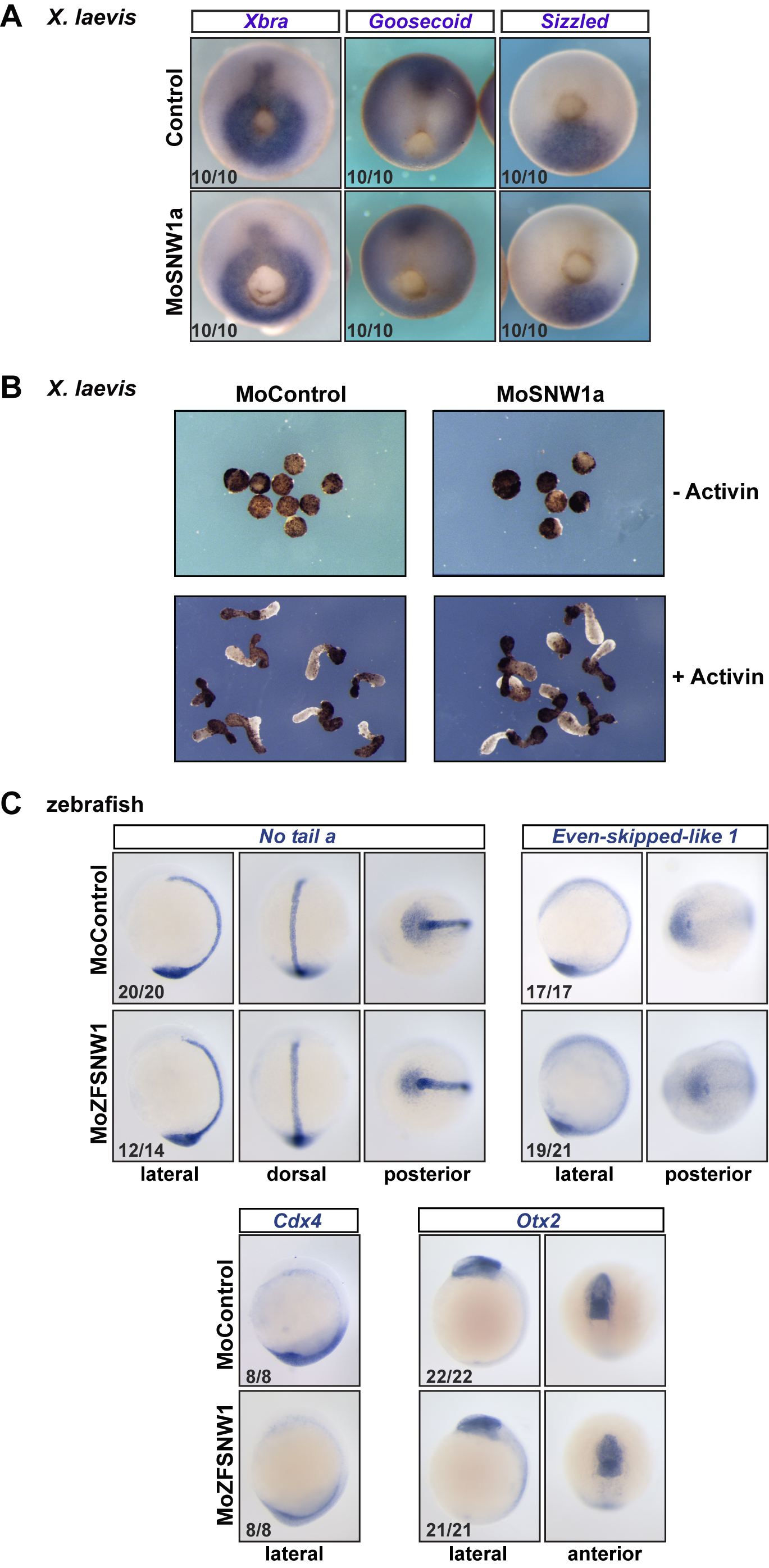

Supplement: Figure S5 — SNW1 knockdown has no effect on mesoderm induction or gastrulation and does not affect Activin-dependent induction of mesodermal tissue in Xenopus animal caps. (A) One-cell Xenopus embryos were injected with 20 ng of MoSNW1a. Uninjected control and MoSNW1a-injected embryos were fixed at stage 12 for WISH using probes against Xbra, Goosecoid, and Sizzled. (B) Animal caps dissected from MoSNW1a-injected embryos elongate similarly in response to 20 ng/ml Activin (PeproTech) as caps cut from control embryos. Thus mesoderm induction is not affected by injection of MoSNW1a. (C) Zebrafish embryos were injected with 15 ng of either control MO or MoZFSNW1. The embryos were fixed at 12 hpf and stained for the mesoderm markers No tail a (axial and tail), Even-skipped-like 1 (paraxial and tail), and Cdx4 (posterior axial and tail as well as some ectoderm). In SNW1 morphants, the expression of the mesoderm markers is largely unchanged, but there is a slight reduction in their expression in the tail mesoderm. Otx2 is a marker for anterior neural ectoderm and is normal albeit slightly expanded in SNW1 morphants. Otx2 and Cdx4 expression also indicate that anterior/posterior patterning is preserved in the absence of SNW1. In all cases the number of embryos out of the total analyzed that showed the presented staining pattern is given. (3.44 MB TIF) [file pbio.1000593.s005.tif]

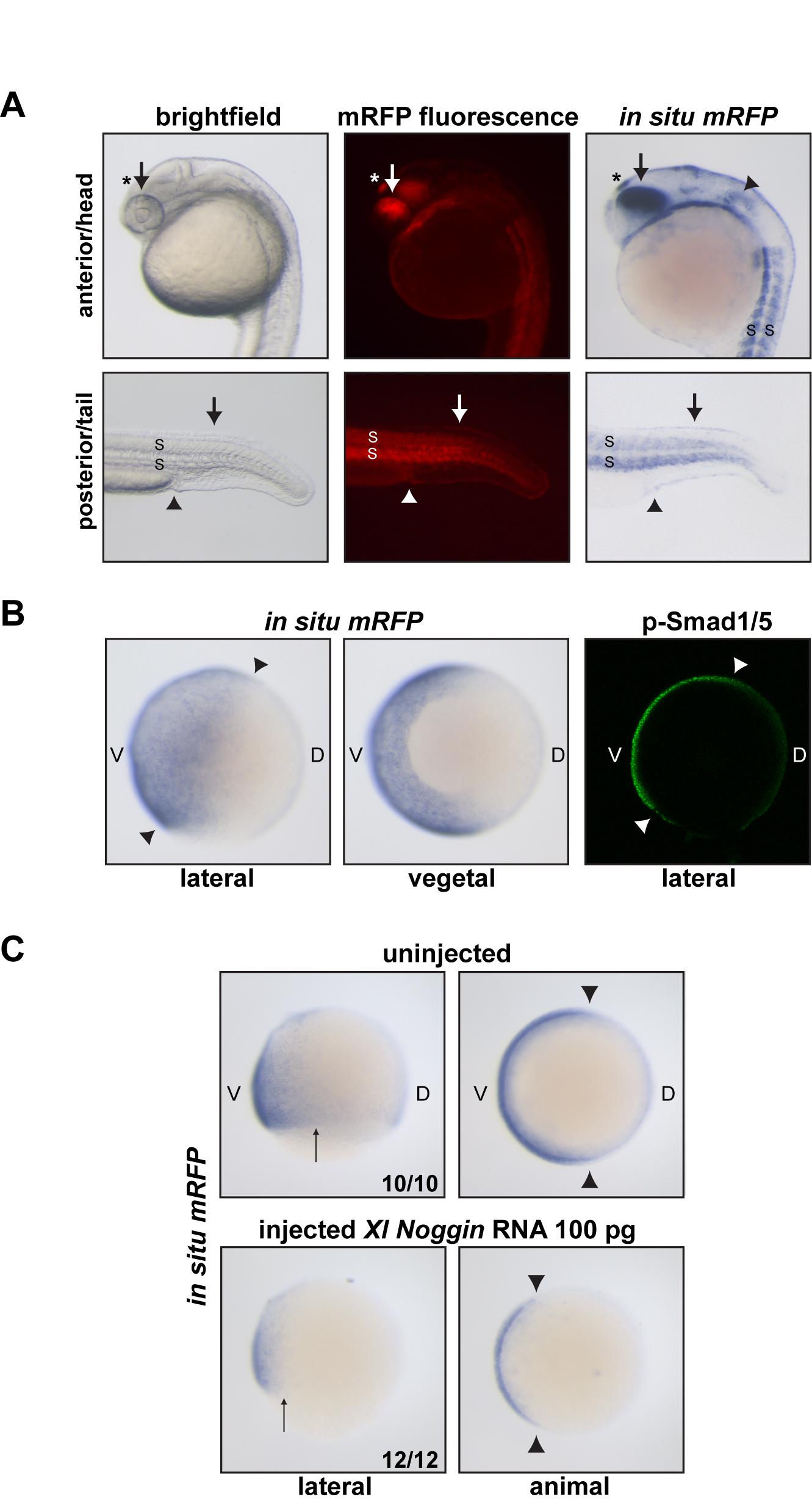

Supplement: Figure S6 — The BRE- mRFP transgenic zebrafish line is a reporter for BMP activity. (A) 24-h transgenic embryos imaged using bright field or fluorescence microscopy, or processed for in situ hybridization using a probe against mRFP. mRFP protein or mRNA is indicative of BMP activity. Indeed, in the anterior/head region, mRFP is detected in known sites of BMP expression and/or activity such as the dorsal retina and lens (arrow; [79]) and the otic placode (arrowhead; [80]). mRFP is also strongly detected in the epiphysis/pineal gland, consistent with data obtained in Xenopus (asterisk; [81]). In the posterior/tail region, mRFP as a readout of BMP signaling is present for instance in the dorsal ectoderm, where BMP ligands are expressed (arrow; [82]), the cloaca (arrowhead; [83]), and the somites (S; [82]). (B) mRFP in situ hybridization on transgenic embryos at 85% epiboly. mRFP transcripts are detected in the ventral ectoderm and the ventral lateral plate and intermediate mesoderm, where BMP signaling is known to be active [26]. The mRFP pattern is consistent with p-Smad1/5 staining at the same stage (arrowheads; see also [4]). (C) Noggin overexpression inhibits mRFP transcription downstream of the BRE promoter. As expected, injection of X. laevis Noggin mRNA results in reduced BMP signaling [26], which leads to lower amounts of mRFP transcripts in 70% epiboly embryos. Arrows and arrowhead indicate the extent of the mRFP expression domain for comparison between uninjected and injected embryos. In (B) and (C), V, ventral; D, dorsal. The number of embryos out of the total analyzed that showed the presented staining pattern is given. (2.69 MB TIF) [file pbio.1000593.s006.tif]

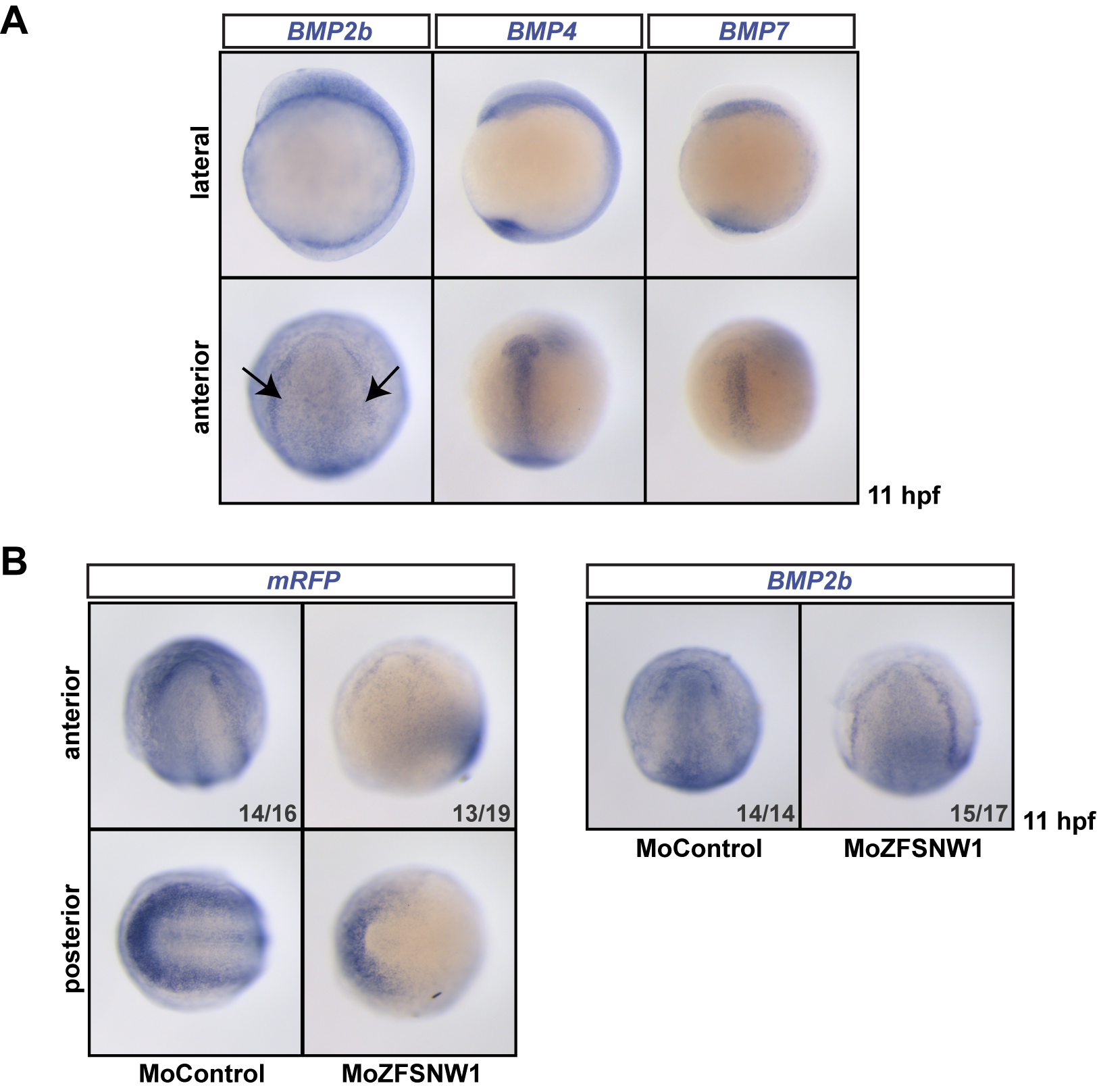

Supplement: Figure S7 — BMP2b is the BMP family member in zebrafish that accounts for the BMP activity at the epidermis/neural ectoderm border that is dependent on SNW1. (A) Comparison of BMP2b, BMP4, and BMP7 expression in zebrafish embryos at 11 hpf (2–3 somite stage) using WISH. BMP2b transcripts are enriched at the epidermis/neural ectoderm border (arrows). In contrast, BMP4 is enriched in the anterior prechordal plate and the tailbud, while BMP7 appears to be expressed in the endoderm. (B) Transgenic BRE-mRFP embryos were injected with 15 ng of control MO or MoZFSNW1. They were fixed at 11 hpf, when WISH was performed for mRFP. SNW1 knockdown results in the strong loss of BMP activity at the epidermis/neural ectoderm border, as seen with mRFP WISH in BRE-mRFP embryos. Notably, some of the posterior mRFP staining is preserved in the SNW1 morphant, which is likely because of BMP4 activity. BMP2b expression, however, appears unaltered in SNW1 morphants, suggesting that SNW1 does not induce BMP activity at the epidermis/neural ectoderm border through the transcriptional regulation of BMP2b. Note that the same batch of injected embryos was stained for mRFP or BMP2b. (2.22 MB TIF) [file pbio.1000593.s007.tif]

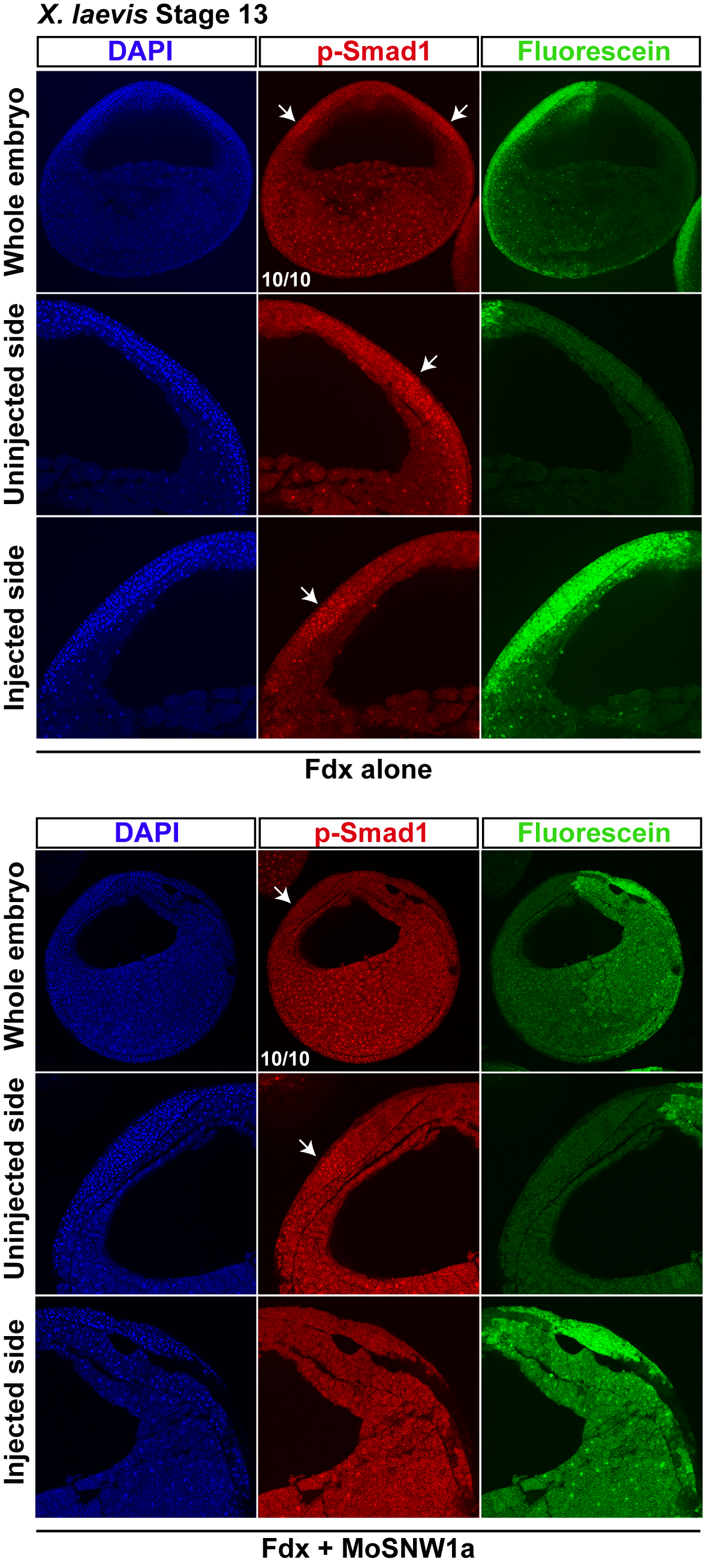

Supplement: Figure S8 — Localized BMP activity is detected at the neural plate border region in Xenopus embryos, and this is dependent on SNW1. Fdx with or without 20 ng of MoSNW1a was injected into one cell of two-cell embryos. They were fixed at stage 13, bisected transversely through the neural crest region, and immunostained with antibodies against p-Smad1 and fluorescein. Nuclei were stained with DAPI. The specific p-Smad1 staining is nuclear and punctate. White arrowheads indicate a domain of p-Smad1 staining in the neural crest region that is lost on the injected side when SNW1 is depleted with the MO. (2.76 MB TIF) [file pbio.1000593.s008.tif]

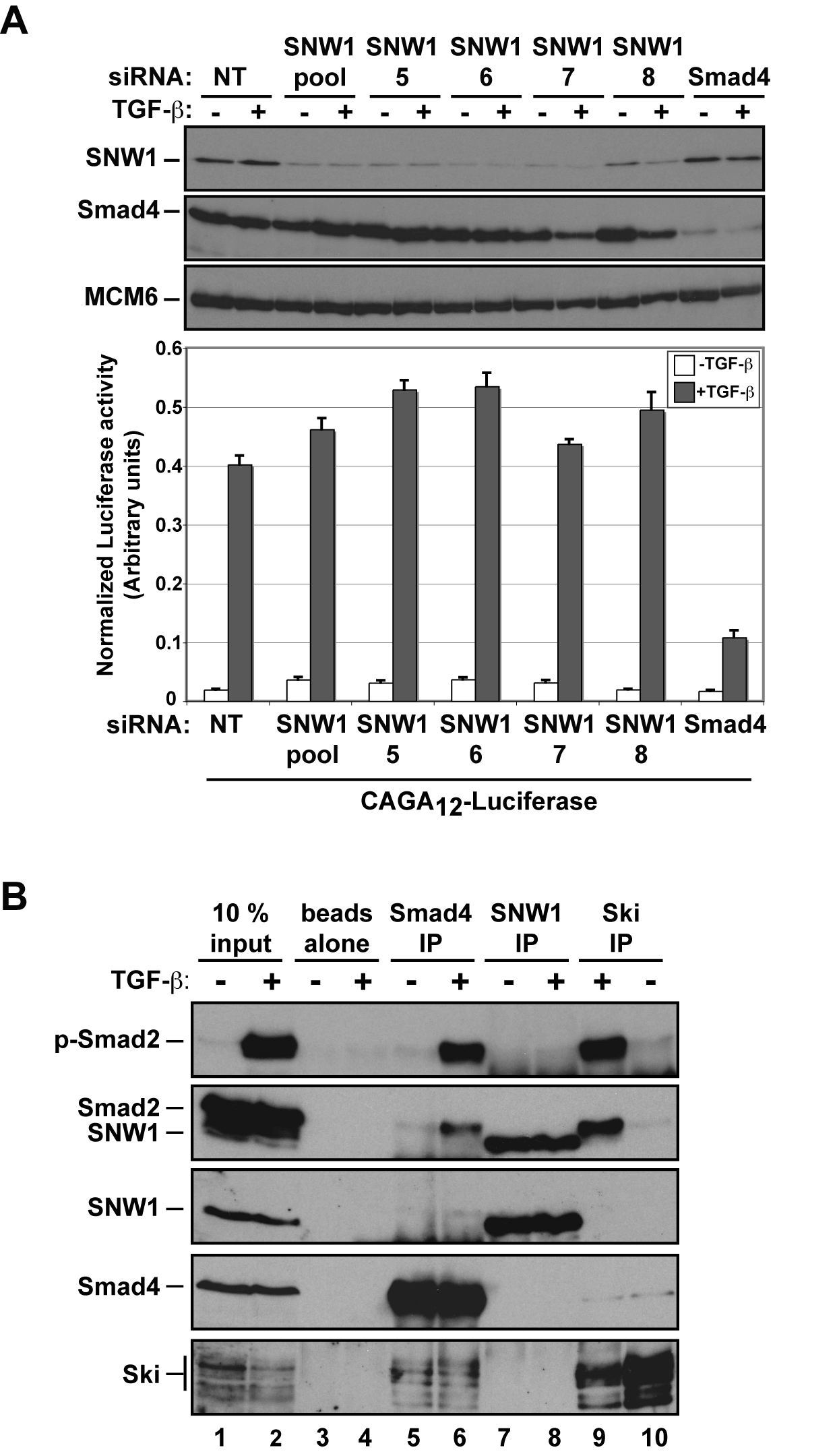

Supplement: Figure S9 — siRNA-mediated knockdown of SNW1 in mammalian cells does not affect TGF-β signaling. (A) siRNA-mediated knockdown of SNW1 in mammalian cells does not affect a TGF-β-responsive reporter activity. An MDA-MB-231 cell line stably expressing TK-Renilla and CAGA12-Luciferase was transfected with a non-targeting siRNA (NT), an siRNA pool against SNW1, the four separate siRNAs that make up the pool, or an siRNA against Smad4. 72 h after transfection, cells were induced with 2 ng/ml TGF-β as indicated. Samples were taken for Western blotting after 1 h and for Luciferase/Renilla assays after 8 h. Whereas knockdown of Smad4 diminishes the CAGA12-Luciferase reporter activity, SNW1 depletion does not significantly alter TGF-β-dependent transcription. The efficiency of knockdown is demonstrated in the Western blots shown. (B) SNW1 does not interact with Ski or Smad proteins at endogenous levels in cells. MDA-MB-231 cells were preincubated for 3 h with 50 µM MG132 (Sigma) to prevent Ski degradation in response to TGF-β, before being induced or not with 2 ng/ml TGF-β for 1 h. Cell were then lysed in 150 mM co-IP buffer and immunoprecipitated with the antibodies indicated. A “beads alone” IP served as a negative control (lanes 3 and 4), and 10% of the total lysate used per IP was reserved for input (lanes 1 and 2). The IPs were blotted with the antibodies indicated. Smad2/3 and p-Smad2 as well as Ski can be co-immunoprecipitated with Smad4, as expected [28], while SNW1 was not co-immunoprecipitated (lanes 5 and 6). Immunoprecipitated SNW1 did not co-immunoprecipitate Smad2/3, Smad4, or Ski (lanes 7 and 8). Only Smad2/3, p-Smad2, and Smad4 co-immunoprecipitated with Ski (lanes 9 and 10). The blot for Smad2/3 is a re-probe of the SNW1 blot, and thus the SNW1 signal can additionally be seen on the Smad2/3 blot. (0.46 MB TIF) [file pbio.1000593.s009.tif]
